# Neuroimaging in lesioning therapy for obsessive-compulsive disorder: region-based and network analysis of preoperative outcome predictors and postoperative effects

**DOI:** 10.1016/j.nicl.2026.103976

**Published:** 2026-02-20

**Authors:** Lyndon Boone, Mahan Shafie, Aariz Naeem, Drew Yang, Jurgen Germann, Yutong Bai, Maged Goubran, Clement Hamani, Nir Lipsman, Victor M. Tang, Alexandre Boutet, Benjamin Davidson

**Affiliations:** a Faculty of Medicine, Memorial University of Newfoundland, St. John’s, NL, Canada; b Sunnybrook Research Institute, Toronto, ON, Canada; c Department of Neuroscience, University of Turin, Turin, Italy; d School of Interdisciplinary Science, Faculty of Science, McMaster University, Hamilton, ON, Canada; e Division of Neurosurgery, Department of Surgery, University of Toronto, Toronto, ON, Canada; f Krembil Brain Institute, Toronto Western Hospital, University Health Network, Toronto, ON, Canada; g Center for Advancing Neurotechnological Innovation to Application, Toronto, ON, Canada; h Department of Neurosurgery, Beijing Tiantan Hospital, Beijing, China; i Harquail Centre for Neuromodulation, Hurvitz Brain Sciences Program, Sunnybrook Research Institute, Toronto, ON, Canada; j Physical Sciences, Sunnybrook Research Institute, Toronto, ON, Canada; k Department of Medical Biophysics, University of Toronto, Toronto, ON, Canada; l Division of Neurosurgery, Sunnybrook Health Sciences Centre, Toronto, ON, Canada; m Addictions Division, Centre for Addiction and Mental Health, Toronto, ON, Canada; n Department of Psychiatry, Temerty Faculty of Medicine, University of Toronto, Toronto, ON, Canada; o Institute for Mental Health Policy Research, Centre for Addiction and Mental Health, Toronto, ON, Canada; p Department of Medical Imaging, University of Toronto, Toronto, ON, Canada

**Keywords:** Capsulotomy, Cingulotomy, Lesioning, MRI, Neuroimaging, Obsessive-compulsive disorder

## Abstract

**Introduction:**

Lesioning procedures such as capsulotomy and cingulotomy offer therapeutic options for patients with severe, treatment-resistant obsessive–compulsive disorder (OCD). However, treatment response remains variable, and mechanisms underlying clinical improvement are poorly understood. Neuroimaging may help identify response predictors and elucidate mechanisms of action.

**Methods:**

We systematically reviewed neuroimaging findings from studies of lesioning therapy for OCD, focusing on preoperative predictors of response and postoperative imaging changes. To synthesize findings, we performed region-based frequency mapping alongside connectivity analyses to characterize the functional networks embedding these regions.

**Results:**

Twenty-four studies met inclusion criteria. Six reported preoperative neuroimaging findings associated with clinical response. Across heterogeneous modalities and metrics, the dorsal anterior cingulate cortex was most frequently implicated in predictive findings. Twenty studies reported postoperative changes, with frequent reductions in volume, metabolism, and connectivity noted in the prefrontal cortex, striatum, and thalamus. Less consistent changes were reported in widespread cortical regions and the cerebellum. Symptom reduction did not significantly differ across affected areas. Connectivity analyses revealed that the insula, paracingulate, and middle temporal gyrus may serve as central nodes within the network linking regions of postoperative change.

**Conclusion:**

Despite high methodological heterogeneity and small sample sizes in the primary literature, these findings underscore the potential of neuroimaging to inform patient selection and clarify mechanisms of OCD lesioning therapy. Network analyses may provide a framework for considering novel therapeutic targets. Future prospective research should verify the role of the anterior cingulate cortex in predicting treatment response, and further explore mechanistic changes in distributed brain circuits.

## Highlights

• Preoperative imaging predictors of OCD lesioning response are in their infancy.
• The dACC is frequently implicated in predictive findings, warranting further study.
• Postoperative imaging consistently suggests decreased fronto-striatal activity.
• Widespread cortical & cerebellar changes beyond CSTC circuits remain underexplored.
• Normative connectivity reveals central network hubs after lesioning.

## 1 Introduction

Obsessive-compulsive disorder (OCD) is a prevalent psychiatric condition characterized by intrusive obsessions or compulsions that lead to significant distress and functional impairment (American Psychiatric Association, 2013, Jalal et al., 2023, Robbins et al., 2019 Apr, National Institute of Mental Health, 2025, Fawcett et al., 2020). Importantly, comorbidity with other psychiatric conditions – particularly major depressive disorder (MDD) – is common, with lifetime rates estimated as high as 50–60% (Denys et al., 2004 Jun 1, Fineberg et al., 2007 May), contributing to increased morbidity and mortality (Mrazek et al., 2014 Aug, Torres, et al., 2025). Although first-line treatments, including pharmacotherapy and cognitive-behavioural therapy, are effective for many, up to one-third of individuals exhibit treatment-resistance (Hirschtritt et al., 2017). For patients with severe, treatment-resistant OCD, neurosurgical lesioning therapy – including radiofrequency (RF) ablation, gamma knife (GK) radiosurgery, laser interstitial thermal therapy (LITT), and magnetic resonance-guided focused ultrasound (MRgFUS) – offers a therapeutic alternative.

Lesioning therapies for OCD generally aim to interrupt hyperactive frontal cortico-striatal-thalamo-cortical (CSTC) circuits implicated in OCD, either through invasive (e.g., RF ablation) or non-invasive lesioning techniques (e.g., GK radiosurgery, MRgFUS) (Barrios-Anderson A, McLaughlin NCR. Obsessive-Compulsive Disorder: Lesions. In: Pouratian N, Sheth SA, editors. Stereotactic and Functional Neurosurgery: Principles and Applications [Internet]. Cham: Springer International Publishing;, 2020, Greenberg et al., 2010 Jan). The most common procedures – capsulotomy and cingulotomy – target the anterior limb of the internal capsule (ALIC) and the cingulum bundle at the level of the anterior cingulate cortex (ACC), respectively. Although there is currently no consensus on selection criteria, patients are typically selected based on having long-standing, severe OCD, measured by standardized assessment tools such as the Yale-Brown Obsessive-Compulsive Scale (Y-BOCS), and not responding to multiple trials of guideline-concordant therapy. Systematic reviews and meta-analyses have demonstrated favorable safety profiles and response rates ranging from 55 to 73% for capsulotomy (Gupta et al., 2024 Mar 29, Lai et al., 2020 Sep, Brown et al., 2016 Jan 1, Pepper et al., 2019 Oct 11) and 36–37% for cingulotomy (Lai et al., 2020 Sep, Brown et al., 2016 Jan 1), where response rate is typically defined as the percentage of patients achieving a Y-BOCS reduction ≥ 35% after treatment.

While response rates to cingulotomy and capsulotomy are favourable, considering the severity and refractoriness of patients being treated, the variability in response, ranging from near-complete remission to minimal benefit, limits the effectiveness of these interventions. This heterogeneity underscores the lack of reliable preoperative predictors of response and reflects a limited understanding of the underlying mechanisms of action (Davidson et al., 2020). Although deep brain stimulation offers a reversible and titratable means of modulating these circuits, current meta-analyses have not demonstrated its superiority to traditional lesioning procedures, and it remains more technically complex and costly (Barrios-Anderson A, McLaughlin NCR. Obsessive-Compulsive Disorder: Lesions. In: Pouratian N, Sheth SA, editors. Stereotactic and Functional Neurosurgery: Principles and Applications [Internet]. Cham: Springer International Publishing;, 2020, Pepper et al., 2015 May 1, Hageman et al., 2021).

Neuroimaging provides a promising avenue for identifying preoperative markers that predict clinical response to lesioning procedures, and for uncovering postoperative changes that may elucidate mechanisms of action. Extensive neuroimaging research has revealed alterations in brain metabolism, structure, and connectivity in individuals with OCD compared to healthy controls – particularly within frontal CSTC circuits involving the orbitofrontal cortex (OFC) and the ACC (Saxena and Rauch, 2000). Importantly, OCD is a heterogeneous disorder, with symptom dimensions such as symmetry/ordering, contamination/cleaning, checking, and hoarding (now classified as a distinct entity) representing clinically meaningful subtypes (Mataix-Cols et al., 2005 Feb, Mataix-Cols et al., 2004 Jun 1, Saxena et al., 2004 Jun, An et al., 2009 Mar, van den Heuvel et al., 2009 Apr 1). These subtypes have not only shown differing responses to lesioning interventions in some studies (Ruck et al., 2012, Gong et al., 2019 Nov 29, Baer, 1995 May 1), but emerging evidence also suggests they may be associated with distinct neuroimaging signatures – raising the possibility that neuroimaging biomarkers could help guide patient selection. Neuroimaging has also been widely employed to investigate the structural and functional effects of lesioning procedures (Saxena and Rauch, 2000). Although numerous studies report changes across widespread cortical and subcortical regions postoperatively, most prevailing models of capsulotomy and cingulotomy for OCD continue to emphasize CSTC circuits involving the caudate, OFC, and ACC. This highlights a need to synthesize findings across the literature to identify converging patterns of brain activity and connectivity that may serve as a shared neural substrate underlying treatment response.

In this work, we review neuroimaging findings from lesioning studies in OCD to address two key challenges. First, we examine preoperative imaging features associated with positive treatment outcomes in capsulotomy and cingulotomy, aiming to inform strategies for improved patient selection. Second, we compile evidence of postoperative neuroimaging changes following capsulotomy and cingulotomy, aiming to identify common brain regions affected by treatment, and whether specific regional changes correlate with clinical response. We performed region-based frequency mapping alongside connectivity analyses to characterize the functional networks which embed these regions. By integrating these findings, we seek to advance our understanding of the neuroanatomical mechanisms underlying lesioning therapies and lay the groundwork for imaging-informed approaches to patient selection.

## 2 Materials and methods

### 2.1 Search strategy and data sources

We performed a systematic review of the literature following PRISMA guidelines (PROSPERO: CRD42024623711). The search strategy centered on two primary concepts: (1) lesional therapies for OCD and (2) neuroimaging. This strategy was refined to ensure comprehensive coverage of relevant literature. Searches were conducted on July 5, 2024, across MEDLINE, Embase, and Scopus since their inception date (Supplementary Tables S1-S3).

### 2.2 Eligibility criteria

Studies were included if they investigated neuroimaging findings in OCD patients who underwent lesioning therapy – including RF ablation, GK radiosurgery, LITT, MRgFUS, capsulotomy, cingulotomy, limbic leucotomy, and subcaudate tractotomy. Eligible studies used magnetic resonance imaging (MRI), functional MRI (fMRI), diffusion tensor imaging (DTI), positron emission tomography (PET), or single-photon emission computed tomography (SPECT) preoperatively to assess response predictors or postoperatively to evaluate treatment-related changes. In addition to standard structural and functional neuroimaging modalities, we included magnetoencephalography (MEG) and electroencephalography (EEG) studies that met our inclusion criteria due to their potential to complement neuroimaging findings. For postoperative changes, we only included studies that compared pre- and postoperative imaging within the same individuals, excluding studies that reported postoperative imaging findings with no preoperative comparison, as these could not be confidently attributed to lesioning therapy. We excluded case reports, review articles, and commentaries.

### 2.3 Data management and selection process

All references were imported into Covidence (https://www.covidence.org/) for duplicate removal, title/abstract screening, and full-text review. Two independent reviewers (MS, AN) screened all records and conducted full-text reviews using the predefined eligibility criteria. Disagreements were resolved by a third reviewer (BD).

### 2.4 Data extraction

Two reviewers independently extracted data, resolving discrepancies through consensus. Extracted data included study identification (author, publication year), sample demographics, intervention type and target, key imaging findings, and clinical outcomes. For each study, statistically significant imaging findings were tabulated and mapped to closest regions of interest (ROIs) based on anatomical coordinates or reported structures, using the Harvard-Oxford (Makris et al., 2006 Apr 1, Frazier et al., 2005 Jul, Desikan et al., 2006 Jul 1, Goldstein et al., 2007 Apr 15), Johns Hopkins University (JHU) DTI white matter (Mori et al., 2006 Jun 1, Wakana et al., 2007 Jul 1, Hua et al., 2008 Jan 1), and probabilistic cerebellar atlases included with the FSL library (Diedrichsen et al., 2009 May 15, Jenkinson et al., 2012 Aug 15). Each ROI was categorized by whether it was part of a predictor-related (i.e., preoperative biomarker) or mechanism-related (i.e., postoperative change) finding. For each postoperative finding, ROIs were additionally classified according to the direction of change – either an increase or decrease – in volume or glucose metabolism when applicable (Table 1). For studies that reported group-level pre- and postoperative Y-BOCS scores, we recorded the mean absolute Y-BOCS reduction for clinical outcome mapping (Goodman et al., 1989 Nov 1, Goodman et al., 1989 Nov 1).

**Table 1:** Rules for assigning “increased” or “decreased” labels to postoperative changes for each ROI.

	**−1**	**+1**
**Volume**	Decreased	Increased
**Glucose metabolism**	Decreased	Increased

### 2.5 Synthesis

We generated composite brain maps to aggregate and visualize ROI involvement using three strategies:

1. *Frequency-weighted ROI maps:* For each ROI, we summed the number of patients across all studies citing it in a significant predictor or mechanism-related finding, independent of imaging modality. This allowed ROIs from larger studies to contribute more heavily to the composite map than those from smaller studies. These maps operate under the assumption that ROIs more frequently implicated across the literature are more likely to represent reliable predictors of treatment response or consistent foci of postoperative change.
2. *Directionality maps*: For postoperative findings only, we aggregated the counts of increased/decreased volumetric and metabolic changes per ROI (Table 1), weighting each by study sample size and normalizing to the range [-1, 1] to produce a “normalized directionality index.” This strategy yielded maps illustrating regions with consistent increases or decreases in these imaging features following lesioning therapy. Directionality analyses were restricted to structural MRI and FDG-PET due to insufficient data for robust synthesis of postoperative fMRI and diffusion MRI findings.
3. *Clinical outcome maps*: For each ROI mentioned in a significant postoperative finding, we averaged the absolute mean Y-BOCS reduction from all studies that implicated that ROI and reported cohort-level pre-/postop scores. We used absolute Y-BOCS change (rather than relative percentage change) to minimize bias, as relative change correlated with baseline Y-BOCS severity (Supplementary Fig. S1).

### 2.6 Connectivity analysis

Each ROI identified in the synthesis maps was used as a seed to generate whole-brain functional connectivity *r*-maps using a normative 3T resting-state fMRI dataset from the Brain Genomics Superstruct Project (https://dataverse.harvard.edu/dataverse/GSP). The resultant *r*-maps reflect the average voxel-to-seed blood-oxygen-level dependent signal correlation across the normative dataset. We derived two types of networks to investigate spatial connectivity patterns underlying the ROI frequency maps:

1. *Internal network*: We calculated a cross-correlation matrix between all ROIs and applied hierarchical clustering to identify patterns of inter-regional connectivity. Graph theory metrics (betweenness, closeness) were computed to identify central nodes potentially relevant to treatment response and postoperative effects, the assumption being that the more central a node is to the rest of the graph, the more important its role is in the network. Due to the limited number of ROIs in the preoperative dataset, this analysis was restricted to ROIs exhibiting postoperative change.
2. *External network*: Voxel-wise *r*-maps for each ROI were converted to *t*-maps, and significantly connected voxels were identified using Bonferroni correction for multiple comparisons (*p*_bonferroni_ < 0.05, whole brain). Each voxel was then assigned a value reflecting the number of ROIs to which it was significantly connected. To isolate regions most commonly connected across implicated ROIs, we thresholded these maps to include only voxels connected to ≥ 50% of all ROIs. The assumption is that more highly connected voxels are more likely to belong to higher-order networks which may not be directly implicated in findings from the literature but which could still be relevant to OCD circuitry.

Internal and external networks were generated separately for ROIs associated with preoperative predictors and postoperative changes. To explore convergent network patterns across lesion targets, we constructed combined networks using ROIs from capsulotomy and cingulotomy studies, as well as capsulotomy-only networks (excluding cingulotomy due to limited data). For analyses involving ROIs exhibiting postoperative change, our connectivity analyses were not intended to model postoperative network reorganization, but rather to characterize the functional architecture of regions that are consistently affected by lesioning. By embedding these regions within a normative connectome, we aimed to characterize pre-lesion networks that are preferentially targeted and disrupted by lesioning.

### 2.7 Statistical methods and computational tools

We used Fisher’s exact test to compare the frequency of ROI involvement across studies (Upton, 1992). Each study was treated as a unit of observation, and ROI presence/absence was modeled as a binary variable. To test whether treatment responses differed significantly by ROI, we used the Kruskal–Wallis H-test to compare mean absolute Y-BOCS reductions for ROIs implicated in at least three studies (Kruskal and Wallis, 1952). No inferential statistical tests were applied to directionality maps; these were intended as descriptive, hypothesis-generating tools. Analyses and figure generation were conducted using Python (Virtanen et al., 2020 Mar, McKinney, 2010, Waskom, 2021 Apr 6). Code and data are available on GitHub at https://github.com/lyndonboone/ocd-lesioning-neuroimaging.git.

## 3 Results

### 3.1 Study characteristics

**Fig. 1:**
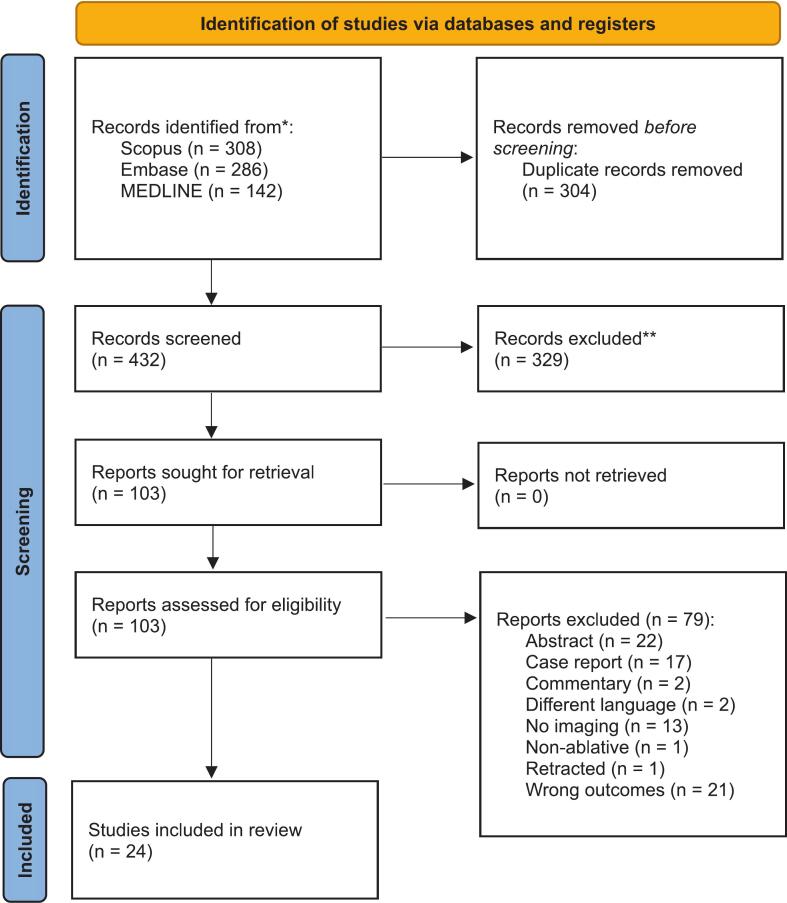
PRISMA flow diagram depicting study selection.

**Fig. 2:**
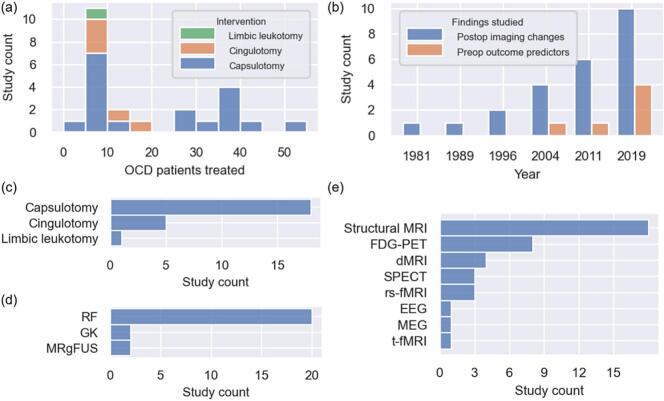
Characteristics of included studies. (a) Number of OCD patients treated and included in the main imaging analyses in individual studies (bars coloured by intervention). (b) Publication year (bars coloured by types of findings studied). (c) Intervention target. (d) Intervention modality. (e) Imaging modalities used. Abbreviations: dMRI, diffusion MRI; EEG, electroencephalography; FDG-PET, fluorodeoxyglucose-positron emission tomography; GK, Gamma Knife; MEG, magnetoencephalography; MRgFUS, magnetic resonance-guided focused ultrasound; MRI, magnetic resonance imaging; OCD, obsessive–compulsive disorder; RF, radiofrequency; rs-fMRI, resting-state functional MRI; SPECT, single-photon emission computed tomography; t-fMRI, task-based functional MRI.

We included 24 studies comprising a total of 443 participants (Fig. 1, Table 2, Table 3). Sample sizes were small (median = 10; range = 3–53; Fig. 2a), and publications spanned from 1978 to 2023 (Fig. 2b). Of the included studies, six examined preoperative neuroimaging findings associated with treatment outcome (Table 2), while 21 reported postoperative neuroimaging changes (Table 3). Capsulotomy (18 studies) and cingulotomy (5 studies) were the most common lesion targets (Fig. 2c), with radiofrequency ablation being the predominant intervention modality (Fig. 2d). Considerable heterogeneity was observed in terms of imaging modalities and experimental design (Fig. 2e). Only one EEG study (Bingley and Persson, 1978) and one MEG study (Chang et al., 2023) met inclusion criteria, limiting the ability to meaningfully integrate these findings with those from other neuroimaging modalities (Table 3).

**Table 2:** Summary of preoperative imaging characteristics which predicted outcome after OCD lesioning therapy, organized by study. ROIs are highlighted in bold font.

**Author (year)**	N*	**Procedure**	**Main findings**
Banks et al. (2015) (Banks et al., 2015)	15	Bilateral RF cingulotomy	*Gray matter:* Responders had decreased GM volume in a cluster just anterior to the lesion area, in the **right paracingulate gyrus**. *White matter:* Responders had greater **right-** than left-sided structural connectivity between the **dACC** lesion area and the ipsilateral **thalamus**, **putamen**, **pallidum**, and **hippocampus**. When a standardized lesion mask was used instead of the patient-specific one, the same findings were present except the **caudate** instead of the putamen was significant.
**Davidson et al. (2020) (Davidson et al., 2020)	6	Bilateral MRgFUS capsulotomy	Responders demonstrated increased preop functional connectivity in three connectivity pairs: **right ventral striatum-right hippocampus; left dorsal putamen-left occipital cortex; left dorsal putamen-left postcentral gyrus**.
Lv et al. (2021) (Lv et al., 2021)	31	Bilateral RF capsulotomy	Responders showed smaller GM volume in the **right IFG**, and lower generalized FA in the **left SLF** and **right cingulum** bundle.
Rauch et al. (2001) (Rauch et al., 2001)	11	Bilateral RF cingulotomy	Higher **right PCC** glucose metabolism was correlated with Y-BOCS improvement.
Yin et al. (2018) (Yin et al., 2018)	36	Bilateral RF capsulotomy	Responders showed decreased functional connectivity between the **dACC** and **dorsal caudate**.
Zhang et al. (2021) (Zhang et al., 2021)	41	Bilateral RF capsulotomy	Increased normalized streamline counts between the **thalamus** and **dorsolateral PFC** (**frontal pole** on HO atlas) and **dorsal ACC** were positively correlated with Y-BOCS improvement.

*Includes lesioned OCD patients who were included in the main imaging analysis.

**These studies featured mixed cohorts, including OCD and MDD patients.

Abbreviations: ACC, anterior cingulate gyrus; dACC, dorsal ACC; FA, fractional anisotropy; GM, gray matter; IFG, inferior frontal gyrus; PCC, posterior cingulate cortex; PFC, prefrontal cortex; RF, radiofrequency; SLF, superior longitudinal fasciculus; Y-BOCS, Yale-Brown Obsessive Compulsive Scale.

**Table 3:** Summary of postoperative imaging changes after OCD lesioning therapy, organized by study. ROIs are highlighted in bold font.

**Author (year)**	N*	**Procedure**	**Main findings**
Bingley and Persson (1978) (Bingley and Persson, 1978)	35	Bilateral RF capsulotomy	75% of patients exhibited **bilaterally** synchronous bursts of rhythmic slow waves at maximum in the **frontal regions** approximately one week after surgery. 79% of patients returned to the same preop EEG pattern after 1–2 years.
Bouwens van der Vlis et al. (2022) (Bouwens van der Vlis et al., 2022)	8	Bilateral GK capsulotomy	Decrease in **left ventral diencephalon** volume (hypothalamus, mammillary bodies, STN, LGN, MGN, red nuclei, substantia nigra) and **right cerebellum white matter** was positively correlated with Y-BOCS improvement. There were no changes in cortical volumes postop. Fiber tracts passing through the lesion which connected the **STN** with **prefrontal cortex** were associated with Y-BOCS improvement.
Cecconi et al. (2008) (Cecconi et al., 2008)	5	Bilateral GK capsulotomy	Increased GM volume in the **right lateral OFC** (BA 47)
Chang et al. (2003) (Chang et al., 2003)	8	Bilateral RF cingulotomy	Reduced perfusion within the **ACC** and **right OFC**
Chang et al. (2023) (Chang et al., 2023)	8	Bilateral MRgFUS capsulotomy	High beta band power in the **fronto-central** and **temporal** areas decreased. This decrease was correlated with anxiety/depression symptom improvement at 1 month, but not at 6 months, despite the fact that clinical improvements persisted at 6 months.
Chen et al. (2022) (Chen et al., 2022)	27	Bilateral RF capsulotomy	In general, there was decreased **cortico-subcortical**, and increased **cortico-cortical** functional connectivity across several ROI pairs post-capsulotomy. Averaged communication strength with other cortical nodes was significantly increased for the **right medial OFC**, **left lateral OFC**, **left ventrolateral PFC**, and **left centrolateral PFC**.
Cui et al. (2023) (Cui et al., 2023)	27	Bilateral RF capsulotomy	*Gray matter:* Post-capsulotomy patients had lower GM volume in **bilateral caudate**, **thalamus**, and **NAcc** compared to OCD controls. *Task-based fMRI:* During the feedback phase of an aversive avoidance task, OCD controls had greater **left ventrolateral PFC** activity to aversive images relative to post-capsulotomy patients and healthy controls. Post-capsulotomy patients had lower **left rACC** activity during extinction feedback relative to OCD and healthy controls (i.e., when subjects expected a negative feedback image but observed a neutral image).
**Davidson et al. (2020) (Davidson et al., 2020)	6	Bilateral MRgFUS capsulotomy	Reduced glucose metabolism: **bilateral middle temporal gyri, pre/post central gyri, right middle frontal gyrus, right PCC, right amygdala, hippocampus, and putamen.**
**Hurwitz et al. (2020) (Hurwitz et al., 2020)	3	Bilateral RF capsulotomy	Decreased rCBF in the **paraterminal gyri**. Increased rCBF in the **DLPFC** and **left lateral temporal lobe**.
Kim et al. (2001) (Kim et al., 2001)	8	Bilateral RF limbic leukotomy	Decreased rCBF in the **right medial frontal cortex** and **left striatum** compared to baseline.
Liu et al. (2008) (Liu et al., 2008)	35	Bilateral RF capsulotomy	Glucose metabolism significantly decreased bilaterally in the **ACC**, **OFC**, and **caudate**.
Liu et al. (2017) (Liu et al., 2017)	37	Bilateral RF capsulotomy	Glucose metabolism significantly decreased bilaterally in the **ACC**, **OFC**, and **caudate**.
Lv et al. (2021) (Lv et al., 2021)	31	Bilateral RF capsulotomy	Widespread decrease in GM volume bilaterally across **frontal** and **temporal cortices**, **cerebellum**, **thalami**, and **caudate** nuclei. Smaller GM volume of the **right caudate** was found in responders compared to non-responders.
Mindus et al. (1990) (Mindus et al., 1990)	5	Bilateral RF capsulotomy	Decreased glucose metabolism bilaterally in the **OFC** and **caudate**.
Rauch et al. (2000) (Rauch et al., 2000)	9	Bilateral RF cingulotomy	Bilateral decrease in **caudate** volume. No change in thalamus or amygdala volume.
Rauch et al. (2001) (Rauch et al., 2001)	9	Bilateral RF cingulotomy	Decreased GM volume in **right PCC** and **left posterior temporal fusiform gyrus**.
Suetens et al. (2014) (Suetens et al., 2014)	13	Bilateral RF capsulotomy	*Decreased glucose metabolism:***Left caudate** and **ACC**, **bilateral posterior cerebellum**, **right inferior frontal cortex**. *Increased glucose metabolism:***Right occipital (fusiform/lingual) gyri, left cerebellum (anterior lobe), left parietal, postcentral gyrus, and precuneus, right angular gyrus and precuneus, and right middle temporal gyrus**.
Taren et al. (1994) (Taren et al., 1994)	5	Bilateral RF capsulotomy	Decreased **caudate** and **thalamus** volume.
Yin et al. (2018) (Yin et al., 2018)	36	Bilateral RF capsulotomy	Decreased functional connectivity between **dACC** and **ventral striatum** (correlated with Y-BOCS change), and **dACC** and **putamen**. Responders additionally had decreased functional connectivity between the **medial OFC** and **ventral striatum**.
Zhan et al. (2014) (Zhan et al., 2014)	53	Bilateral RF capsulotomy	Decreased glucose metabolism bilaterally in **ACC**, **OFC**, and **caudate**.
Zuo et al. (2013) (Zuo et al., 2013)	8	Bilateral RF capsulotomy	Decreased glucose metabolism bilaterally in prefrontal cortical regions, particularly the **dorsal ACC**, medial dorsal **thalamus**, and **caudate**. Increased metabolism bilaterally in **precentral** and **lingula gyri**.

*Includes lesioned OCD patients who were included in the main imaging analysis.

**These studies featured mixed cohorts, including OCD and MDD patients without OCD.

Abbreviations: ACC, anterior cingulate gyrus; CBF, cerebral blood flow; dACC, dorsal ACC; DLPFC, dorsolateral PFC; EEG, electroencephalography; FA, fractional anisotropy; GM, gray matter; IFG, inferior frontal gyrus; LGN, lateral geniculate nucleus; MGN, medial geniculate nucleus; OCD, obsessive–compulsive disorder; OFC, orbitofrontal cortex; PCC, posterior cingulate cortex; PFC, prefrontal cortex; rACC, rostral ACC; rCBF, regional CBF; RF, radiofrequency; SLF, superior longitudinal fasciculus; STN, subthalamic nucleus; Y-BOCS, Yale-Brown Obsessive Compulsive Scale.

### 3.2 Preoperative outcome predictors

**Fig. 3:**
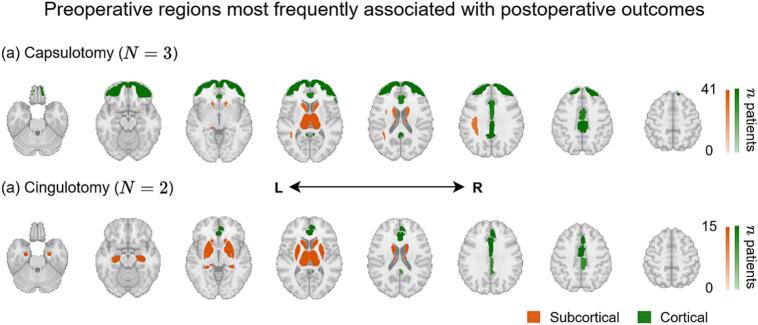
Preoperative regions most frequently reported to be associated with postoperative outcomes after (a) capsulotomy and (b) cingulotomy. Brain maps display the number of patients (*n*) summed across studies that included each ROI in a preoperative finding. One study was excluded from these maps as its cohort combined OCD and MDD patients (50). Abbreviations: L, left; R, right.

Six studies reported preoperative neuroimaging features associated with treatment response following capsulotomy or cingulotomy (Table 2, Fig. 3). In an RF capsulotomy study, responders exhibited reduced gray matter volume in the right inferior frontal gyrus (IFG) (*p* < 0.05), as well as lower fractional anisotropy in the left superior longitudinal fasciculus and right cingulum (*p* < 0.05) (Lv et al., 2021). Other studies highlighted connectivity-based markers, with responders showing reduced functional connectivity between the dorsal ACC (dACC) and dorsal caudate (*R*^2^ = 0.23, *p* = 0.011) (Yin et al., 2018), or conversely, increased structural connectivity (based on normalized DTI streamline counts) between the thalamus and frontal regions, including the dACC (*r* = 0.34, *p* = 0.0402) and frontal pole (*r* = 0.36, *p* = 0.0347) (Zhang et al., 2021). A separate MRgFUS capsulotomy study that included both OCD and MDD patients found that clinical improvement was associated with increased functional connectivity in circuits involving the ventral striatum, hippocampus, dorsal putamen, occipital cortex, and postcentral gyrus (Davidson et al., 2020).

Cingulotomy studies also noted structural and metabolic differences between responders and non-responders. One study reported smaller gray matter volume in the right paracingulate gyrus in responders (0.47; units not provided) vs. non-responders (0.66) (*p* < 0.05), along with greater right- than left-sided structural connectivity from the lesion site in the dACC to subcortical regions including the thalamus, putamen, pallidum, and hippocampus (*p* = 0.001) (Banks et al., 2015). Another study found that higher baseline glucose metabolism in the right posterior cingulate cortex (PCC) correlated with greater Y-BOCS improvement following RF cingulotomy (*p* < 0.01) (Rauch et al., 2001).

### 3.3 Postoperative imaging changes

**Fig. 4:**
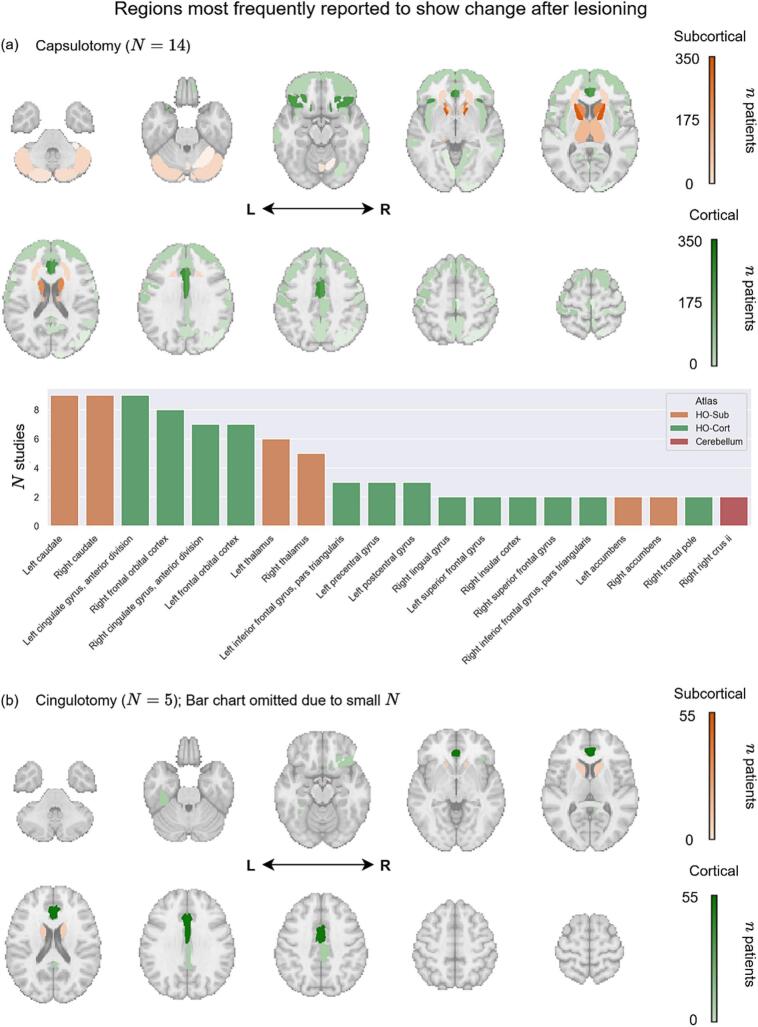
Postoperative changes after (a) capsulotomy and (b) cingulotomy. Brain maps display the number of patients (*n*) summed across studies that included each ROI in a significant finding. Cortical ROIs are shown in green; subcortical ROIs (including the cerebellum) are shown in orange. The bar chart in (a) displays the number of studies (*N*) that included each ROI in a significant finding for *N* > 1. Abbreviations: HO-Cort, Harvard-Oxford cortical atlas; HO-Sub, Harvard-Oxford subcortical atlas; L, left; R, right. (For interpretation of the references to colour in this figure legend, the reader is referred to the web version of this article.)

Twenty studies were included in the image synthesis analysis, excluding those with mixed psychiatric cohorts (e.g., OCD and MDD without OCD) (Davidson et al., 2020, Hurwitz et al., 2020), or those using EEG/MEG (Bingley and Persson, 1978, Chang et al., 2023). Across capsulotomy studies, the most commonly reported ROIs were the bilateral caudate nuclei, ACC, OFC, and thalamus (Fig. 4a; Supplementary Table S4). Using Fisher’s exact test, we found no statistically significant differences in the frequency of these ROIs in postoperative findings (all pairwise *p* > 0.25), nor any lateralized differences in involvement between hemispheres. In addition to these primary ROIs, the left IFG, precentral gyrus, and postcentral gyrus were each identified in three studies. Several other ROIs spanning all four cortical lobes, subcortical regions, and the cerebellum were each reported in two or fewer studies (Fig. 4a; Supplementary Table S4).

Few studies examined postoperative imaging changes following cingulotomy (Table 3). Findings included reduced perfusion in the right OFC and decreased volume in the bilateral caudate nuclei, right PCC, and left posterior temporal fusiform gyrus (Fig. 4b, Supplementary Table S5).

### 3.4 Directionality maps

**Fig. 5:**
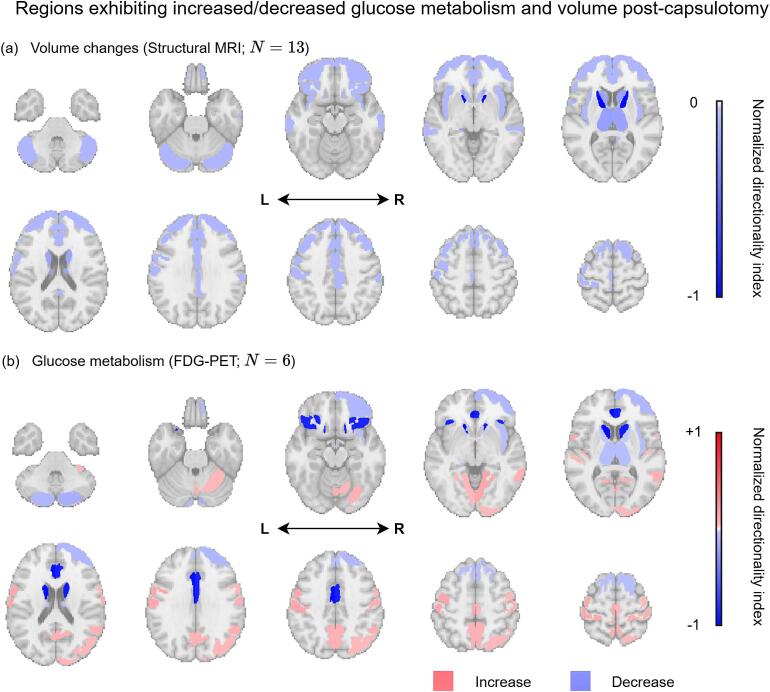
Directionality maps displaying regions reported to exhibit increased/decreased (a) volume and (b) glucose metabolism post-capsulotomy. Red and blue colours indicate postoperative increases or decreases, respectively (Table 1). Abbreviations: FDG-PET, fluorodeoxyglucose-positron emission tomography; L, left; MRI, magnetic resonance imaging; R, right. (For interpretation of the references to colour in this figure legend, the reader is referred to the web version of this article.)

Postoperative directionality patterns were examined separately by imaging modality in capsulotomy studies due to limited data for cingulotomy. Structural MRI studies (*n* = 13) consistently demonstrated volume reductions in prefrontal cortex, caudate nuclei, thalamus, and cerebellum (Fig. 5a; Supplementary Table S6). FDG-PET studies (*n* = 6) revealed a more heterogeneous metabolic pattern, characterized by reduced glucose metabolism in frontal regions alongside relative increases in posterior sensorimotor and association cortices, including the precentral and postcentral gyri, precuneus, angular gyrus, and middle temporal gyrus (Fig. 5b; Supplementary Table S7). Both increased and decreased metabolism were reported in cerebellar regions (Suetens et al., 2014, Zuo et al., 2013).

### 3.5 Clinical outcome mapping

**Fig. 6:**
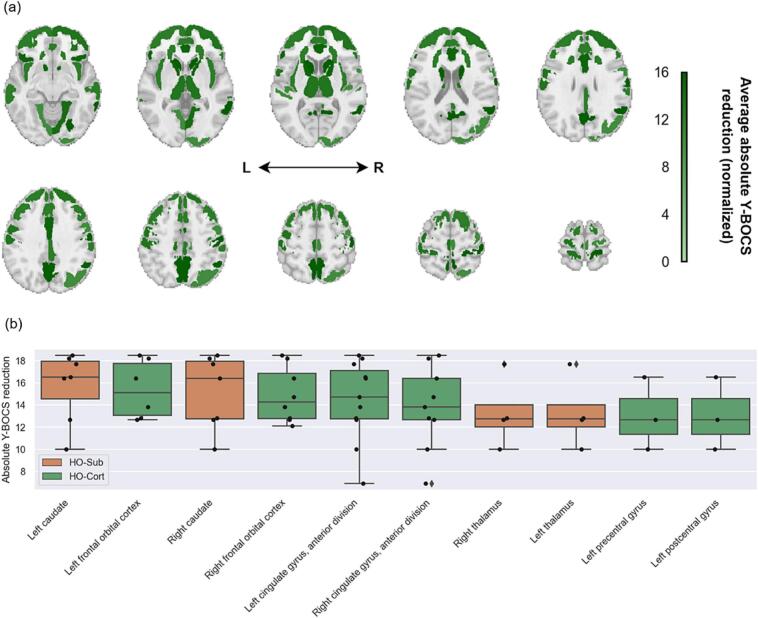
(a) Average absolute postoperative Y-BOCS reduction across capsulotomy and cingulotomy studies that included each ROI in a significant finding. The average was weighted by the number of subjects in each study. (b) Absolute Y-BOCS reduction across cortical and subcortical ROIs mentioned in at least three studies. Abbreviations: HO-Cort, Harvard-Oxford cortical atlas; HO-Sub, Harvard-Oxford subcortical atlas; L, left; R, right; Y-BOCS, Yale-Brown Obsessive-Compulsive Score.

To explore the relationship between imaging findings and clinical outcomes, we analyzed 14 studies that reported mean preoperative and postoperative Y-BOCS scores. Mean absolute Y-BOCS reductions across ROIs were relatively consistent (range: 13.1–15.7), and no statistically significant differences were detected in the distribution of clinical response across ROIs (Kruskal-Wallis H-test, *p* = 0.82; Fig. 6). This finding remained unchanged when limiting the analysis to capsulotomy studies alone (*n* = 11; *p* = 0.78; Supplementary Fig. S2).

### 3.6 Connectivity analysis

**Fig. 7:**
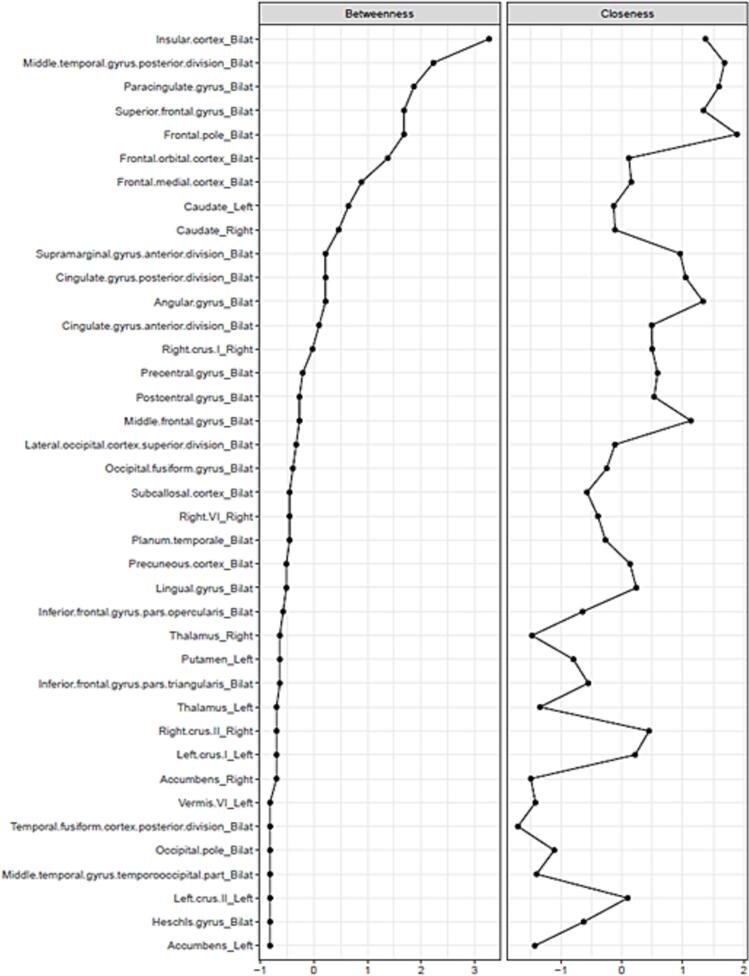
Betweenness and closeness metrics of each ROI included in the internal network, comprised of ROIs which demonstrated postoperative change after capsulotomy and cingulotomy. Abbreviations: Bilat, bilateral.

The internal network for ROIs identified from postoperative changes after capsulotomy and cingulotomy demonstrated central nodes (based on betweenness) that weren’t prominently featured in the frequency-weighted ROI maps, including the insula, middle temporal gyrus, paracingulate cortex, superior frontal gyrus (SFG), and frontal pole (Fig. 7). Excluding cingulotomy studies, the central nodes of the internal network remained the same except the OFC became the most central node in the network (Supplementary Fig. S3).

**Fig. 8:**
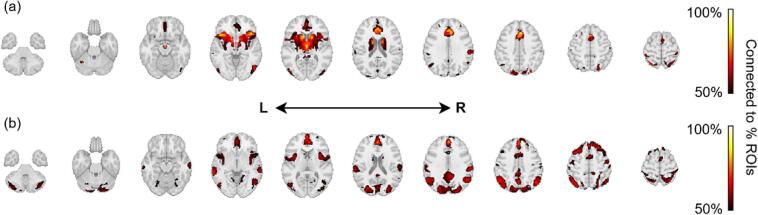
Whole brain regions most frequently functionally connected to the ROIs included in frequency-weighted maps for (a) preoperative predictors and (b) postoperative changes combining capsulotomy and cingulotomy. Abbreviations: L, left; R, right; ROIs, regions-of-interest.

The external network identified the regions most functionally connected to those identified in our frequency-based ROI maps. Most voxels in the external network derived from preoperative predictor ROIs were restricted to frontal CSTC regions including the basal ganglia, thalamus, IFG, ACC, and paracingulate cortex (Fig. 8a). Meanwhile, voxels in the external network derived from postoperative change ROIs spanned more diffuse regions across the cortex including the somatosensory cortex, precuneus, and inferior parietal lobe, in addition to more frontal regions including the ACC, paracingulate, IFG, and SFG (Fig. 8b). These trends were largely preserved after excluding cingulotomy studies (Supplementary Fig. S4).

## 4 Discussion

Lesioning therapy for OCD remains challenging due to the high variability in clinical response, underscoring a need for optimized patient selection and improved understanding of therapeutic mechanisms of action. This work aimed to synthesize neuroimaging findings from OCD lesioning studies to better understand treatment response predictors and mechanisms underlying improvement. While preoperative neuroimaging findings varied across studies, aberrations in several regions – including the ACC, striatum, thalamus, and other prefrontal areas – were repeatedly implicated as predictors of response, suggesting potential convergence on robust biomarkers. Meanwhile, postoperative findings consistently demonstrated changes in frontostriatal and thalamic regions, with more sporadic changes reported beyond frontal CSTC circuits.

Neuroimaging predictors of treatment success should be interpreted in the context of other clinical factors known to influence treatment response. Studies have suggested that OCD symptom dimensions may predict differential responses to lesioning, bolstering the idea of distinct clinical subtypes with unique neurobiological substrates. For example, capsulotomy appears less effective for patients with prominent symmetry/ordering or hoarding symptoms (Ruck et al., 2012, Gong et al., 2019 Nov 29), whereas cingulotomy may yield greater benefit in this group (Baer, 1995). Neuroimaging research supports the existence of such subtypes, particularly in hoarding-predominant OCD, which has been associated with reduced metabolism in the dorsal ACC, PCC, and cuneus and increased ventromedial prefrontal cortex activity during symptom provocation (Saxena et al., 2004 Jun, An et al., 2009 Mar). Some regions implicated in these studies, especially the dACC, were also found in our preoperative predictors (Yin et al., 2018, Zhang et al., 2021, Banks et al., 2015). However, methodological differences – such as variability in connectivity measures and lesion targets – make it challenging to link these findings. These findings highlight a key gap: few studies have jointly analyzed symptom dimensions, preoperative imaging, and treatment outcomes in the same cohort. Such integrative approaches may be essential for developing robust, clinically useful predictors.

Interestingly, some seemingly conflicting findings in the ‘prediction’ data may be reconciled by considering which networks are directly interrupted by the lesion. Zhang et al. found that greater structural connectivity (normalized streamline counts) between the thalamus and both the dlPFC and dACC – tracts directly passing through the ALIC − predicted better response to capsulotomy, suggesting that responders require a sufficient preoperative ‘substrate’ (Zhang et al., 2021). In contrast, Lv et al. showed that lower white matter integrity (generalized fractional anisotropy) in the superior longitudinal fasciculus and cingulum – tracts well outside the lesion area − predicted better outcomes, possibly reflecting vulnerability in more distal cognitive control networks (Lv et al., 2021). Yin et al. reported that reduced functional connectivity between the dorsal caudate and dACC – regions connected by fibers slightly dorsal to the capsulotomy lesions − predicted response (Yin et al., 2018). Taken together, these findings suggest that stronger connectivity in tracts traversing the lesion site may be necessary for effective disruption, while weaker integrity in distant control networks may independently predict better response. These patterns may differ in cingulotomy, where the lesion directly targets the dACC and cingulum.

Postoperative imaging changes after lesioning therapy revealed more consistent patterns, with many reporting decreased volume or metabolism in frontostriatal regions following capsulotomy and cingulotomy. Across modalities, findings commonly implicated the caudate, ACC, OFC, and thalamus. These regions align with well-established models of OCD pathophysiology, which posit hyperactivity within frontal CSTC loops (Robbins et al., 2019 Apr, Jalal et al., 2023, Graybiel and Rauch, 2000 Nov 1, Ahmari et al., 2013). When stratified by modality, structural MRI studies demonstrated consistent reductions in volume across frontostriatal regions, whereas FDG-PET studies showed a more heterogeneous pattern, characterized by reduced frontal metabolism alongside relatively increased metabolism in posterior sensorimotor and association cortices, with mixed results in the cerebellum. This dissociation suggests that frontal changes may reflect a core effect of lesioning on CSTC circuitry, while posterior metabolic increases are less likely to represent structural change and may instead reflect redistribution of neural activity or downstream network-level effects. These regions beyond frontal networks display functional and structural differences in OCD patients compared to healthy controls, with numerous studies citing differences in the cerebellum in particular (Nabeyama et al., 2008 Aug 30, Tobe et al., 2010, Shobeiri et al., 2024 Apr 1, Zhang et al., 2019, Narayanaswamy et al., 2016 Aug, Anticevic et al., 2014 Apr 15, Xu et al., 2019 May, Hartmann et al., 2016 Jun, Tonna et al., 2014 Aug 1), but their role in lesioning-related improvement remains uncertain. These findings highlight the importance of a global network approach involving the entire brain in future lesioning studies searching for therapeutic mechanisms of action.

Recent computational and biophysical modeling studies provide a useful mechanistic framework for interpreting these findings. Virtual intervention models have predicted that symptom improvement in OCD may arise from reducing coupling between the nucleus accumbens and orbitofrontal cortex, while strengthening coupling between lateral prefrontal regions and the dorsal putamen (Naze et al., 2025). Consistent with these predictions, several studies included in this review demonstrated reduced functional connectivity between the OFC and ventral striatum following capsulotomy (Yin et al., 2018, Chen et al., 2022). In contrast, none of the included studies reported increased connectivity between lateral prefrontal cortex and dorsal putamen, suggesting that this component of the modeled therapeutic mechanism may not be consistently engaged by capsulotomy and could represent a complementary or unmet target for intervention. Additionally, recent work has proposed that normalization of functional connectivity gradients within the basal ganglia may underlie symptom improvement in OCD (Webb et al., 2025). While our review confirms that basal ganglia structures are among the most frequently affected regions following lesioning therapy, none of the included studies explicitly examined gradient-level reorganization. Incorporating such approaches in future lesioning and neuromodulation studies may help bridge empirical imaging findings with emerging mechanistic models of OCD circuitry.

Despite the widespread findings across studies, there were no significant differences in symptom reduction across regions found to undergo postoperative change. This lack of association may reflect the coarse spatial resolution of ROI-based analyses, the averaging of heterogeneous symptom profiles within study cohorts, or variability in imaging modalities, timing, and analysis methods. It is also possible that symptom relief depends on the modulation of distributed networks rather than focal changes in isolated regions. The absence of region-specific associations provides a reminder of the possibility that many postoperative imaging changes may represent downstream or epiphenomenal network effects (e.g., diaschisis) rather than direct mechanisms of symptom improvement. Future work may benefit from a *meta*-analysis of clinical outcomes at the individual patient level, which would reveal significantly greater variation in response compared to cohort-wide means. Such work requires a collaborative, shared clinical/imaging repository, which is conceivable given the few centers regularly performing ablative procedures for OCD.

Normative connectivity analysis offers a more nuanced understanding than traditional ROI-based approaches. Graph theory analysis of the internal network constructed from ROIs exhibiting postoperative change highlighted the insula, paracingulate cortex, and frontal pole as central nodes – despite not appearing frequently in ROI-based analyses (Fig. 4). These regions may represent central components of the canonical networks disrupted by lesioning, even if they are not as frequently reported as sites of direct postoperative change. In light of the growing emphasis on modulating large-scale brain networks rather than isolated regions (Horn, 2021, Siddiqi et al., 2021 Dec), these regions may also represent novel avenues for therapeutic exploration, both in lesioning approaches and potentially as future ‘multiple-target’ neuromodulation targets. The external network analysis revealed regions in the frontal, parietal, and somatosensory cortices frequently connected to ROIs that demonstrated postoperative changes. While these areas were not themselves reported as altered in the included studies, their strong functional connectivity with regions showing postoperative change suggests they may be indirectly involved in broader network dynamics. Although we cannot infer direct modulation or therapeutic relevance, such regions could plausibly participate in downstream effects via their network relationships.

This review has several important limitations. First, the included studies were highly heterogeneous in imaging modalities, lesion targets, and analytic approaches, making it difficult to synthesize results quantitatively. Sample sizes were small, limiting statistical power and increasing the risk of false positives or spurious associations. In addition, many studies reported ROIs without precise anatomical coordinates or standardized atlases, requiring approximation during aggregation. ROIs from the chosen atlases were large, resulting in loss of spatial specificity; however, their size allowed for adequate data pooling. Some studies may have focused exclusively on frontal ROIs based on *a priori* hypotheses about OCD circuitry, introducing selection bias and potentially resulting in false negatives in the posterior cortices and cerebellum. While our connectivity analyses did not establish direct statistical associations between network properties and clinical outcomes, they provided a framework for identifying regions not yet significantly implicated in the literature that may warrant future investigation. Importantly, the use of a normative connectome models the canonical network architecture targeted and disrupted by lesioning, rather than the reorganized postoperative connectome. While this approach enabled generalized network mapping, it does not account for individual variability in functional architecture or disruption to postoperative networks, particularly in patients with severe, treatment-resistant OCD treated by lesioning.

## 5 Conclusion

Neuroimaging studies of OCD lesioning therapy reveal a growing but fragmented body of evidence highlighting potential biomarkers of treatment response and mechanisms of therapeutic change. While promising findings have emerged, substantial variability across studies limits generalizability. Widespread regions beyond frontal CSTC circuits may play a role in the pathophysiology of OCD and therapeutic mechanisms of action of lesioning therapy, although this line of research is still in its infancy. Future work should prioritize standardized imaging protocols, larger sample sizes, and integration of clinical phenotyping with imaging data. Multisite collaborations and prospective longitudinal designs, particularly those linking preoperative imaging features to postoperative symptom trajectories, will be essential in translating neuroimaging insights into clinical tools that can guide patient selection and optimize outcomes.

## CRediT authorship contribution statement

**Lyndon Boone:** Writing – review & editing, Writing – original draft, Visualization, Software, Methodology, Investigation, Formal analysis, Data curation, Conceptualization. **Mahan Shafie:** Writing – review & editing, Writing – original draft, Methodology, Investigation, Conceptualization. **Aariz Naeem:** Writing – review & editing, Methodology, Investigation. **Drew Yang:** Writing – review & editing, Methodology, Investigation. **Jurgen Germann:** Writing – review & editing, Software, Methodology, Formal analysis. **Yutong Bai:** Writing – review & editing, Software, Formal analysis. **Maged Goubran:** Writing – review & editing, Supervision. **Clement Hamani:** Writing – review & editing, Supervision. **Nir Lipsman:** Writing – review & editing, Supervision. **Victor M. Tang:** Writing – review & editing, Supervision, Methodology, Investigation, Conceptualization. **Alexandre Boutet:** Writing – review & editing, Writing – original draft, Visualization, Supervision, Software, Project administration, Methodology, Investigation, Formal analysis, Conceptualization. **Benjamin Davidson:** Writing – review & editing, Writing – original draft, Supervision, Project administration, Methodology, Investigation, Conceptualization.

## Funding

This research did not receive any specific grant from funding agencies in the public, commercial, or not-for-profit sectors.

## Declaration of Competing Interest

The authors declare that they have no known competing financial interests or personal relationships that could have appeared to influence the work reported in this paper.

## Data Availability

All of our code will be made available on GitHub at https://github.com/lyndonboone/ocd-lesioning-neuroimaging.git

## References

[b0005] Ahmari S.E., Spellman T., Douglass N.L., Kheirbek M.A., Simpson H.B., Deisseroth K. (2013). Repeated Cortico-Striatal Stimulation Generates Persistent OCD-Like Behavior. Science.

[b0010] American Psychiatric Association (2013).

[b0015] An S.K., Mataix-Cols D., Lawrence N.S., Wooderson S., Giampietro V., Speckens A. (2009 Mar). To discard or not to discard: the neural basis of hoarding symptoms in obsessive-compulsive disorder. Mol. Psychiatry.

[b0020] Anticevic A., Hu S., Zhang S., Savic A., Billingslea E., Wasylink S. (2014 Apr 15). Global resting-state functional magnetic resonance imaging analysis identifies frontal cortex, striatal, and cerebellar dysconnectivity in obsessive-compulsive disorder. Biol. Psychiatry.

[b0025] Baer L. (1995 May 1). Cingulotomy for intractable obsessive-compulsive disorder: prospective long-term follow-up of 18 patients. Arch. Gen. Psychiatry.

[b0030] Banks G.P., Mikell C.B., Youngerman B.E., Henriques B., Kelly K.M., Chan A.K. (2015). Neuroanatomical characteristics associated with response to dorsal anterior cingulotomy for obsessive-compulsive disorder. JAMA Psychiat..

[b0035] Barrios-Anderson A, McLaughlin NCR. Obsessive-Compulsive Disorder: Lesions. In: Pouratian N, Sheth SA, editors. Stereotactic and Functional Neurosurgery: Principles and Applications [Internet]. Cham: Springer International Publishing; 2020 [cited 2025 Apr 13]. p. 445–56. Available from: https://doi.org/10.1007/978-3-030-34906-6_30.

[b0040] Bingley T., Persson A. (1978). EEG studies on patients with chronic obsessive-compulsive neurosis before and after psychosurgery (stereotaxic bilateral anterior capsulotomy). Electroencephalogr. Clin. Neurophysiol..

[b0045] Bouwens van der Vlis T.A.M., Samanci Y., Ackermans L., Schruers K.R.J., Temel Y., Leentjens A.F.G., et al. Network analysis in Gamma Knife capsulotomy for intractable obsessive-compulsive disorder. Brain and Spine. 2022;2((Bouwens van der Vlis, Ackermans, Temel) Department of Neurosurgery, Maastricht University Medical Centre, Maastricht, Netherlands(Samanci, Peker) Department of Neurosurgery, School of Medicine, Koc University, Istanbul, Turkey(Ackermans, Schruers, Temel,):100892. 10.1016/j.bas.2022.100892 PMC9562250 36248148

[b0050] Brown L.T., Mikell C.B., Youngerman B.E., Zhang Y., McKhann G.M., Sheth S.A. (2016 Jan 1). Dorsal anterior cingulotomy and anterior capsulotomy for severe, refractory obsessive-compulsive disorder: a systematic review of observational studies. J. Neurosurg..

[b0055] Cecconi J.P., Lopes A.C., Duran F.L.D.S., Santos L.C., Hoexter M.Q., Gentil A.F. (2008). Gamma ventral capsulotomy for treatment of resistant obsessive-compulsive disorder: a structural MRI pilot prospective study. Neurosci. Lett..

[b0060] Chang J.G., Kim D.-W., Jung H.H., Chang W.S., Kim C.-H., Kim S.J., et al. Evaluation of changes in neural oscillation after bilateral capsulotomy in treatment refractory obsessive-compulsive disorder using magnetoencephalogram. Asian Journal of Psychiatry. 2023;82((Chang) Department of Psychiatry, Myongji Hospital, Hanyang University College of Medicine, Goyang, South Korea(Kim) School of Healthcare and Biomedical Engineering, Chonnam National University, Yeosu, South Korea(Jung, Chang, Chang) Department of Neurosu):103473. 10.1016/j.ajp.2023.103473 36706511

[b0065] Chang J.W., Kim C.H., Lee J.D., Chung S.S. (2003). Single photon emission computed tomography imaging in obsessive-compulsive disorder and for stereotactic bilateral anterior cingulotomy. Neurosurg. Clin. N. Am..

[b0070] Chen X., Lv Q., van Wingen G., Fridgeirsson E.A., Denys D., Voon V. (2022). Common and differential connectivity profiles of deep brain stimulation and capsulotomy in refractory obsessive-compulsive disorder. Mol. Psychiatry.

[b0075] Cui H., Zhang Y., Zhao Y., Ding Q., Chen R., Manssuer L. (2023). Mechanisms underlying capsulotomy for refractory obsessive-compulsive disorder: neural correlates of negative affect processing overlap with deep brain stimulation targets. Mol. Psychiatry.

[b0080] Davidson B., Hamani C., Rabin J.S., Goubran M., Meng Y., Huang Y. (2020). Magnetic resonance-guided focused ultrasound capsulotomy for refractory obsessive compulsive disorder and major depressive disorder: clinical and imaging results from two phase I trials. Mol. Psychiatry.

[b0085] Davidson B., Suresh H., Goubran M., Rabin J.S., Meng Y., Mithani K. (2020 Nov). Predicting response to psychiatric surgery: a systematic review of neuroimaging findings. J. Psychiatry Neurosci..

[b0090] Denys D., Tenney N., van Megen H.J.G.M., de Geus F., Westenberg H.G.M. (2004 Jun 1). Axis I and II comorbidity in a large sample of patients with obsessive–compulsive disorder. J. Affect. Disord..

[b0095] Desikan R.S., Ségonne F., Fischl B., Quinn B.T., Dickerson B.C., Blacker D. (2006 Jul 1). An automated labeling system for subdividing the human cerebral cortex on MRI scans into gyral based regions of interest. Neuroimage.

[b0100] Diedrichsen J., Balsters J.H., Flavell J., Cussans E., Ramnani N. (2009 May 15). A probabilistic MR atlas of the human cerebellum. Neuroimage.

[b0110] Fawcett. E.J., Power, H., Fawcett, J.M., 2020. Women Are at Greater Risk of OCD Than Men: A Meta-Analytic Review of OCD Prevalence Worldwide. J Clin Psychiatry [Internet]. 2020 Jun 23 [cited 2025 Mar 21];81(4). Available from: https://www.psychiatrist.com/jcp/ocd-prevalence-and-gender. 10.4088/JCP.19r13085 32603559

[b0115] Fineberg N.A., Saxena S., Zohar J., Craig K.J. (2007 May). Obsessive-Compulsive Disorder: Boundary Issues. CNS Spectr..

[b0120] Frazier J.A., Chiu S., Breeze J.L., Makris N., Lange N., Kennedy D.N. (2005 Jul). Structural Brain magnetic Resonance Imaging of Limbic and Thalamic Volumes in Pediatric Bipolar Disorder. AJP.

[b0125] Goldstein J.M., Seidman L.J., Makris N., Ahern T., O’Brien L.M., Caviness V.S. (2007 Apr 15). Hypothalamic abnormalities in schizophrenia: sex effects and genetic vulnerability. Biol. Psychiatry.

[b0130] Gong F., Li B., Zhang S., Wang Y., Gao Y., Xu Y. (2019 Nov 29). The suitability of different subtypes and dimensions of obsessive-compulsive disorder for treatment with anterior capsulotomy: a long-term follow-up study. Stereotact. Funct. Neurosurg..

[b0135] Goodman W.K., Price L.H., Rasmussen S.A., Mazure C., Fleischmann R.L., Hill C.L. (1989 Nov 1). The Yale-Brown Obsessive Compulsive Scale: I. Development, use, and Reliability. Arch. Gen. Psychiatry.

[b0140] Goodman W.K., Price L.H., Rasmussen S.A., Mazure C., Delgado P., Heninger G.R. (1989 Nov 1). The Yale-Brown Obsessive Compulsive Scale: II. Validity. Archives of General Psychiatry..

[b0145] Graybiel A.M., Rauch S.L. (2000 Nov 1). Toward a Neurobiology of Obsessive-Compulsive Disorder. Neuron.

[b0150] Greenberg B.D., Rauch S.L., Haber S.N. (2010 Jan). Invasive Circuitry-based Neurotherapeutics: Stereotactic Ablation and Deep Brain Stimulation for OCD. Neuropsychopharmacol.

[b0155] Gupta R., Chen J.W., Hughes N.C., Hamo M., Jean-Baptiste S., Paulo D.L. (2024 Mar 29). Benefits of stereotactic radiosurgical anterior capsulotomy for obsessive-compulsive disorder: a meta-analysis. J. Neurosurg..

[b0160] Hageman S.B., van Rooijen G., Bergfeld I.O., Schirmbeck F., de Koning P., Schuurman P.R. (2021). Deep brain stimulation versus ablative surgery for treatment-refractory obsessive-compulsive disorder: a meta-analysis. Acta Psychiatr. Scand..

[b0165] Hartmann T., Vandborg S., Rosenberg R., Sørensen L., Videbech P. (2016 Jun). Increased fractional anisotropy in cerebellum in obsessive–compulsive disorder. Acta Neuropsychiatrica..

[b0170] Hirschtritt M.E., Bloch M.H., Mathews C.A. (2017 Apr 4). Obsessive-Compulsive Disorder: advances in Diagnosis and Treatment. J. Am. Med. Assoc..

[b0175] Horn A. (2021). Connectomic Deep Brain Stimulation. Academic Press.

[b0180] Hua K., Zhang J., Wakana S., Jiang H., Li X., Reich D.S. (2008 Jan 1). Tract probability maps in stereotaxic spaces: analyses of white matter anatomy and tract-specific quantification. Neuroimage.

[b0185] Hurwitz T.A., Honey C.R., McLeod K.R., Poologaindran A., Kuan A.J. (2020). Hypoactivity in the paraterminal gyrus following bilateral anterior capsulotomy. Can. J. Psychiatry.

[b0190] Jalal B., Chamberlain S.R., Sahakian B.J. (2023). Obsessive-compulsive disorder: Etiology, neuropathology, and cognitive dysfunction. Brain and Behavior..

[b0195] Jenkinson M., Beckmann C.F., Behrens T.E.J., Woolrich M.W., Smith S.M. (2012 Aug 15). FSL. Neuroimage..

[b0200] Kim M.-C., Lee T.-K., Son B.-C., Choi C.R., Lee C. (2001). Regional cerebral blood flow changes in patients with intractable obsessive compulsive disorders treated by limbic leukotomy. Stereotact. Funct. Neurosurg..

[b0205] Kruskal W.H., Wallis W.A. (1952). Use of Ranks in One-Criterion Variance Analysis. J. Am. Stat. Assoc..

[b0210] Lai Y., Wang T., Zhang C., Lin G., Voon V., Chang J. (2020 Sep). Effectiveness and safety of neuroablation for severe and treatment-resistant obsessive–compulsive disorder: a systematic review and meta-analysis. Journal of Psychiatry & Neuroscience : JPN..

[b0215] Liu K., Zhang H., Liu C., Guan Y., Lang L., Cheng Y. (2008). Stereotactic treatment of refractory obsessive compulsive disorder by bilateral capsulotomy with 3 years follow-up. J. Clin. Neurosci..

[b0220] Liu H.B., Zhong Q., Wang W. (2017). Bilateral anterior capsulotomy for patients with refractory obsessive-compulsive disorder: a multicenter, long-term, follow-up study. Neurol. India.

[b0225] Lv Q., Yin D., Zhang C., Sun B., Voon V., Wang Z. (2021). Neuroanatomical Substrates and Predictors of Response to Capsulotomy in Intractable Obsessive-Compulsive Disorder. Biol. Psychiatry: Cognit. Neurosci. Neuroimaging.

[b0230] Makris N., Goldstein J.M., Kennedy D., Hodge S.M., Caviness V.S., Faraone S.V. (2006 Apr 1). Decreased volume of left and total anterior insular lobule in schizophrenia. Schizophr. Res..

[b0235] Mataix-Cols D., Wooderson S., Lawrence N., Brammer M.J., Speckens A., Phillips M.L. (2004 Jun 1). Distinct Neural Correlates of Washing, Checking, and Hoarding SymptomDimensions in Obsessive-compulsive Disorder. Arch. Gen. Psychiatry.

[b0240] Mataix-Cols D., do Rosario-Campos M.C., Leckman J.F. (2005 Feb). A Multidimensional Model of Obsessive-Compulsive Disorder. AJP.

[b0245] McKinney W. Data Structures for Statistical Computing in Python. In Austin, Texas; 2010 [cited 2025 Apr 19]. p. 56–61. Available from: https://doi.curvenote.com/10.25080/Majora-92bf1922-00a.

[b0250] Mindus P., Nyman H., Mogard J., Meyerson B.A., Ericson K. (1990). Frontal lobe and basal ganglia metabolism studied with PET in patients with incapacitating obsessive-compulsive disorder undergoing capsulotomy. Nord. J. Psychiatry.

[b0255] Mori S., Wakana S., Nagae-Poetscher L., Van Zijl P. (2006 Jun 1). MRI Atlas of Human White Matter. Am. J. Neuroradiol..

[b0260] Mrazek D.A., Hornberger J.C., Altar C.A., Degtiar I. (2014 Aug). A Review of the Clinical, Economic, and Societal Burden of Treatment-Resistant Depression: 1996–2013. PS.

[b0265] Nabeyama M., Nakagawa A., Yoshiura T., Nakao T., Nakatani E., Togao O. (2008 Aug 30). Functional MRI study of brain activation alterations in patients with obsessive–compulsive disorder after symptom improvement. Psychiatry Res. Neuroimaging.

[b0270] Narayanaswamy J.C., Jose D., Kalmady S.V., Agarwal S.M., Venkatasubramanian G., Janardhan Reddy Y.C. (2016 Aug). Cerebellar volume deficits in medication-naïve obsessive compulsive disorder. Psychiatry Res. Neuroimaging.

[b0105] National Institute of Mental Health, n.d. Obsessive-compulsive disorder (OCD). National Institute of Mental Health. Available at: https://www.nimh.nih.gov/health/statistics/obsessive-compulsive-disorder-ocd (Accessed: 21 March 2025).

[b0275] Naze S., Hearne L.J., Sanz-Leon P., Robinson C., Hall C.V., Sonkusare S. (2025 Aug 11). Mechanisms and interventions promoting healthy frontostriatal dynamics in obsessive-compulsive disorder. Nat. Commun..

[b0280] Pepper J., Hariz M., Zrinzo L. (2015 May 1). Deep brain stimulation versus anterior capsulotomy for obsessive-compulsive disorder: a review of the literature. J. Neurosurg..

[b0285] Pepper J., Zrinzo L., Hariz M. (2019 Oct 11). Anterior capsulotomy for obsessive-compulsive disorder: a review of old and new literature. J. Neurosurg..

[b0290] Rauch S.L., Kim H., Makris N., Cosgrove G.R., Cassem E.H., Savage C.R. (2000). Volume reduction in the caudate nucleus following stereotactic placement of lesions in the anterior cingulate cortex in humans: a morphometric magnetic resonance imaging study. J. Neurosurg..

[b0295] Rauch S.L., Dougherty D.D., Rees C.G., Cassem E.H., Alpert N.M., Price B.H. (2001). Cerebral metabolic correlates as potential predictors of response to anterior cingulotomy for obsessive compulsive disorder. Biol. Psychiatry.

[b0300] Rauch S.L., Makris N., Rees C.G., Kim H., Cassem E.H., Price B.H. (2001). A magnetic resonance imaging study of regional cortical volumes following stereotactic anterior cingulotomy. CNS Spectr..

[b0305] Robbins T.W., Vaghi M.M., Banca P. (2019 Apr). Obsessive-Compulsive Disorder: Puzzles and prospects. Neuron.

[b0310] Ruck C., Larsson K.J., Mataix-Cols D. (2012). Predictors of medium and long-term outcome following capsulotomy for obsessive-compulsive disorder: one site may not fit all. Eur. Neuropsychopharmacol..

[b0315] Saxena S., Rauch S.L. (2000 Sep 1). Functional neuroimaging and the neuroanatomy of obsessive-compulsive disorder. Psychiatr. Clin. N. Am..

[b0320] Saxena S., Brody A.L., Maidment K.M., Smith E.C., Zohrabi N., Katz E. (2004 Jun). Cerebral Glucose Metabolism in Obsessive-Compulsive Hoarding. AJP.

[b0325] Shobeiri P., Hosseini Shabanan S., Haghshomar M., Khanmohammadi S., Fazeli S., Sotoudeh H. (2024 Apr 1). Cerebellar Microstructural Abnormalities in Obsessive–Compulsive Disorder (OCD): a Systematic Review of Diffusion Tensor Imaging Studies. Cerebellum.

[b0330] Siddiqi S.H., Schaper F.L.W.V.J., Horn A., Hsu J., Padmanabhan J.L., Brodtmann A. (2021 Dec). Brain stimulation and brain lesions converge on common causal circuits in neuropsychiatric disease. Nat. Hum. Behav..

[b0335] Suetens K., Nuttin B., Gabriels L., Van Laere K. (2014). Differences in metabolic network modulation between capsulotomy and deep-brain stimulation for refractory obsessive-compulsive disorder. J. Nucl. Med..

[b0340] Taren J.A., Curtis G.C., Gebarski S.S. (1994). Late local and remote structural changes after capsulotomy for obsessive compulsive disorder. Stereotact. Funct. Neurosurg..

[b0345] Tobe R.H., Bansal R., Xu D., Hao X., Liu J., Sanchez J. (2010). Cerebellar morphology in Tourette syndrome and obsessive-compulsive disorder. Ann. Neurol..

[b0350] Tonna M., Ottoni R., Ossola P., De Panfilis C., Marchesi C. (2014 Aug 1). Late-Onset Obsessive-Compulsive Disorder Associated with Left Cerebellar Lesion. Cerebellum.

[b0355] Torres AR, MD, Ramos-Cerqueira PATA, Ferrão PYA, MD, Fontenelle PLF, et al. Psychiatrist.com. [cited 2025 Mar 21]. Suicidality in Obsessive-Compulsive Disorder: Prevalence and Relation to Symptom Dimensions and Comorbid Conditions. Available from: https://www-psychiatrist-com.qe2a-proxy.mun.ca/jcp/suicidality-obsessive-compulsive-disorder-prevalence/. 10.4088/JCP.09m05651blu 21272513

[b0360] Upton G.J.G. (1992). Fisher’s exact Test. J. R. Stat. Soc. A. Stat. Soc..

[b0365] van den Heuvel O.A., Remijnse P.L., Mataix-Cols D., Vrenken H., Groenewegen H.J., Uylings H.B.M. (2009 Apr 1). The major symptom dimensions of obsessive-compulsive disorder are mediated by partially distinct neural systems. Brain.

[b0370] Virtanen P., Gommers R., Oliphant T.E., Haberland M., Reddy T., Cournapeau D. (2020 Mar). SciPy 1.0: fundamental algorithms for scientific computing in Python. Nat. Methods.

[b0375] Wakana S., Caprihan A., Panzenboeck M.M., Fallon J.H., Perry M., Gollub R.L. (2007 Jul 1). Reproducibility of quantitative tractography methods applied to cerebral white matter. Neuroimage.

[b0380] Waskom M. (2021 Apr 6). seaborn: statistical data visualization. JOSS.

[b0385] Webb L., Hearne L.J., Tian Y.E., Zalesky A., Robinson C., Hall C.V. (2025 Nov 1). Altered Striatal Functional Gradients in Obsessive-Compulsive Disorder. Biol. Psychiatry: Cognit. Neurosci. Neuroimaging.

[b0390] Xu T., Zhao Q., Wang P., Fan Q., Chen J., Zhang H. (2019 May). Altered resting-state cerebellar-cerebral functional connectivity in obsessive-compulsive disorder. Psychol. Med..

[b0395] Yin D., Zhang C., Lv Q., Chen X., Zeljic K., Gong H. (2018). Dissociable Frontostriatal Connectivity: Mechanism and Predictor of the Clinical Efficacy of Capsulotomy in Obsessive-Compulsive Disorder. Biol. Psychiatry.

[b0400] Zhan S., Liu W., Li D., Pan S., Pan Y., Li Y., et al. Long-term follow-up of bilateral anterior capsulotomy in patients with refractory obsessive-compulsive disorder. Clinical Neurology and Neurosurgery. 2014;119((Zhan, Liu, Li, Pan, Pan, Sun) Department of Functional Neurosurgery, Ruijin Hospital, Shanghai Jiao Tong University School of Medicine, Shanghai, China(Li, Lin) Department of Psychiatry, Ruijin Hospital, Shanghai Jiao Tong University School of Medicine,):91–5. 10.1016/j.clineuro.2014.01.009 24635934

[b0405] Zhang H, Wang B, Li K, Wang X, Li X, Zhu J, et al. Altered Functional Connectivity Between the Cerebellum and the Cortico-Striato-Thalamo-Cortical Circuit in Obsessive-Compulsive Disorder. Front Psychiatry [Internet]. 2019 Jul 24 [cited 2025 Apr 19];10. Available from: https://www.frontiersin.orghttps://www.frontiersin.org/journals/psychiatry/articles/10.3389/fpsyt.2019.00522/full. 10.3389/fpsyt.2019.00522 PMC6667674 31396115

[b0410] Zhang C., Kim S.-G., Li J., Zhang Y., Lv Q., Zeljic K. (2021). Anterior limb of the internal capsule tractography: Relationship with capsulotomy outcomes in obsessive-compulsive disorder. J. Neurol. Neurosurg. Psychiatry.

[b0415] Zuo C., Ma Y., Sun B., Peng S., Zhang H., Eidelberg D. (2013). Metabolic imaging of bilateral anterior capsulotomy in refractory obsessive compulsive disorder: an FDG PET study. J. Cereb. Blood Flow Metab..

