# Supplementary material for: Neuroimaging in lesioning therapy for obsessive-compulsive disorder: region-based and network analysis of preoperative outcome predictors and postoperative effects
Source: Neuroimage Clin. 2026 Feb 20;49:103976. doi: 10.1016/j.nicl.2026.103976 (PMC13001058; doi:10.1016/j.nicl.2026.103976)
Supplement: Supplementary Data 1 [file mmc1.docx]

**SUPPLEMENTARY INFORMATION**

**Neuroimaging in lesioning therapy for obsessive-compulsive disorder: region-based and network analysis of preoperative outcome predictors and postoperative effects**

Boone et al.


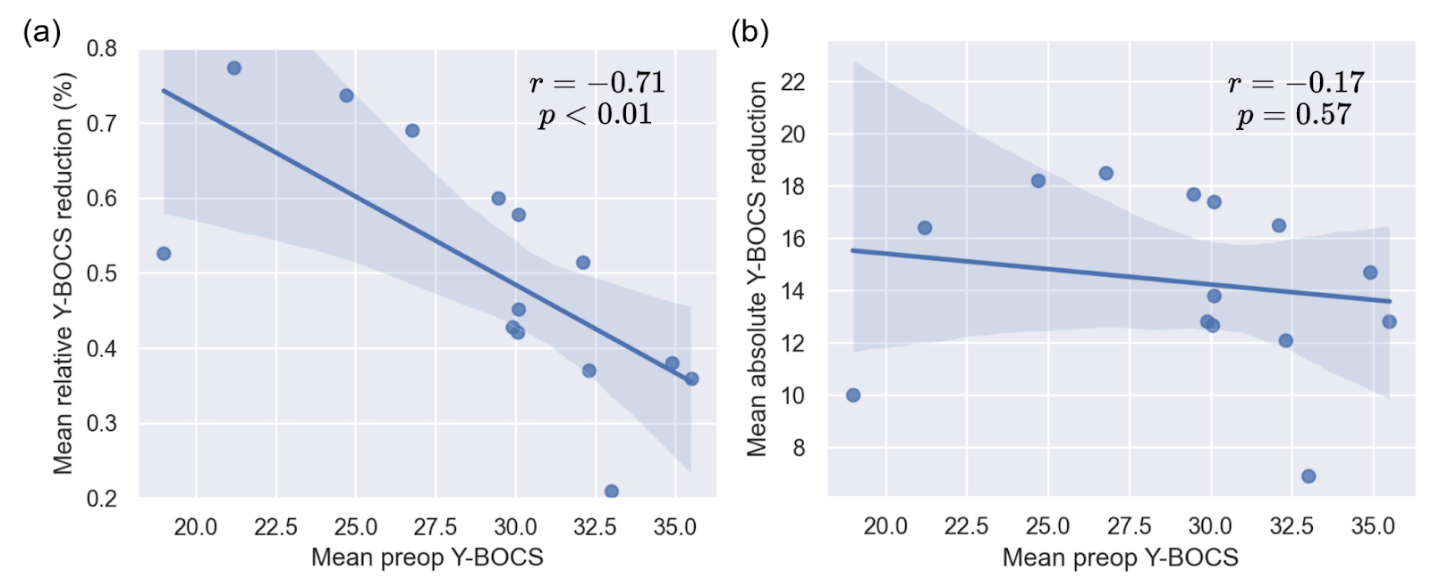


**Supplementary Figure S1:** Correlations between (a) mean relative Y-BOCS reduction (as a percentage of mean preop Y-BOCS score) and (b) mean absolute Y-BOCS reduction (raw score) vs. mean preop Y-BOCS score for 14 capsulotomy and cingulotomy studies which published pre- and postoperative Y-BOCS scores across their cohorts. Shaded bands represent the 95% confidence interval of the regression line, estimated using bootstrapping (1). Abbreviations: *p*, p-value; *r*, Pearson correlation coefficient; Y-BOCS, Yale-Brown Obsessive-Compulsive Scale.


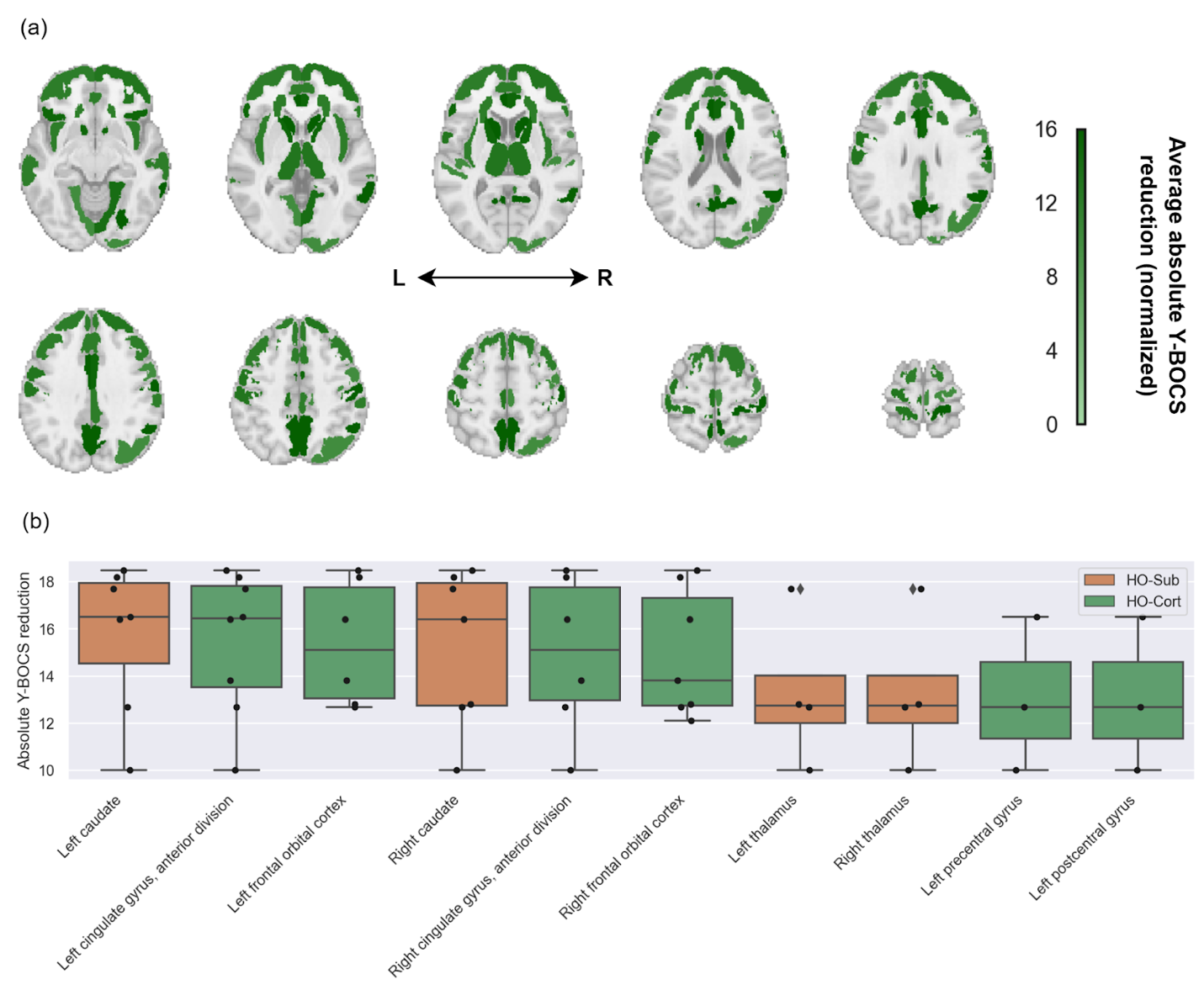


**Supplementary Figure S2:** (a) (a) Average absolute postoperative Y-BOCS reduction across capsulotomy studies that included each ROI in a significant finding. The average was weighted by the number of subjects in each study. (b) Absolute Y-BOCS reduction across cortical and subcortical ROIs mentioned in at least three studies. Abbreviations: HO-Cort, Harvard-Oxford cortical atlas; HO-Sub, Harvard-Oxford subcortical atlas; L, left; R, right; Y-BOCS, Yale-Brown Obsessive-Compulsive Score.


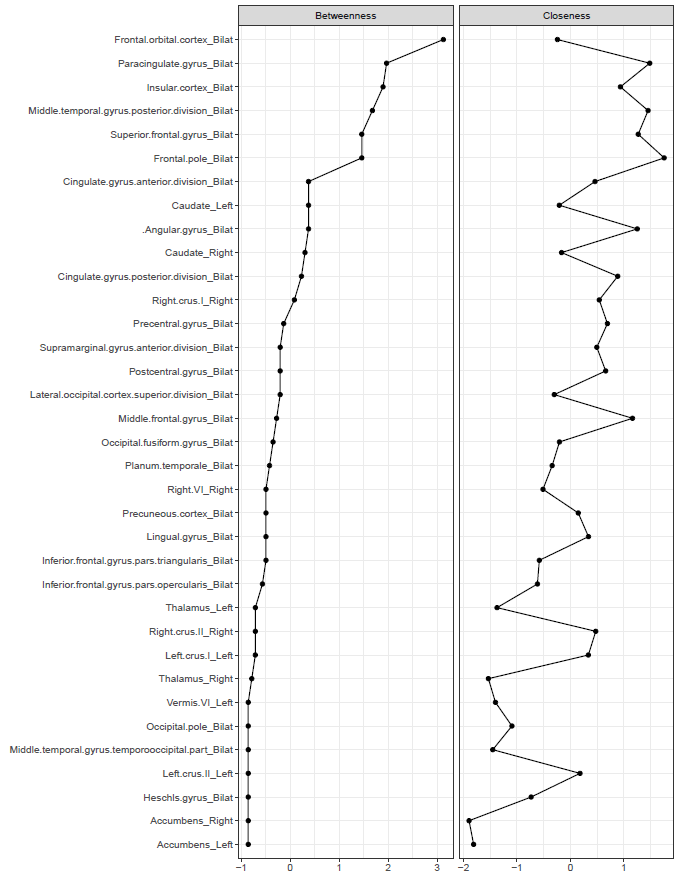


**Supplementary Figure S3:** Betweenness and closeness metrics of each ROI included in the internal network, comprised of ROIs which demonstrated postoperative change after capsulotomy. Abbreviations: Bilat, bilateral.


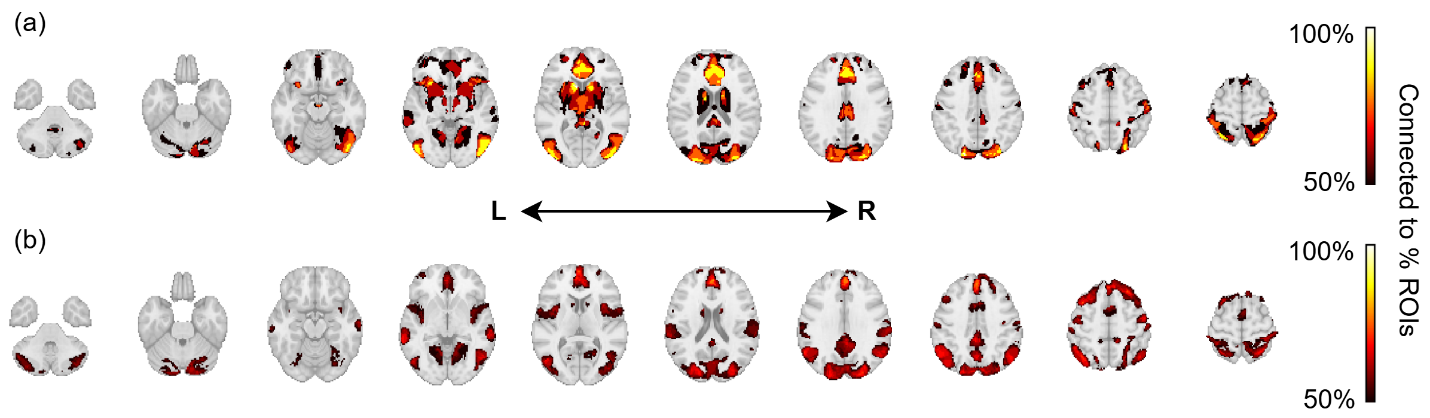


**Supplementary Figure S4**: Whole brain regions most frequently functionally connected to the ROIs included in frequency-weighted maps for (a) preoperative predictors and (b) postoperative changes for capsulotomy only. Abbreviations: L, left; R, right; ROIs, regions-of-interest.

**Supplementary Table S1**: MEDLINE database query, submitted July 5, 2024.

| 1 | obsessive compulsive disorder/ or "OCD".mp. or "obsessive-compulsive disorder".mp. or "obsessive compulsive disorder".mp. | 22445 |
| --- | --- | --- |
| 2 | brain surgery/ or exp psychosurgery/ or exp stereotaxic surgery/ or exp subcaudate tractotomy/ or "psychosurgery".mp. or "gamma knife".mp. or "radiofrequency*".mp. or "cingulotomy".mp. or "capsulotomy".mp. or "*leucotomy".mp. or "*tractotomy".mp. or "MRgFUS".mp. or "focused ultrasound".mp. or "focused-ultrasound".mp. or "gamma knife*".mp. or "GKR".mp. or "cyber knife".mp. or "Laser interstitial thermal therapy".mp. or "LITT".mp. or "*ablative".mp. or "*ablation".mp. | 210479 |
| 3 | tomography, emission-computed/ or positron-emission tomography/ or positron emission tomography computed tomography/ or tomography, emission-computed, single-photon/ or tomography, x-ray computed/ or magnetic resonance imaging/ or diagnostic techniques, neurological/ or magnetoencephalography/ or neuroimaging/ or "*neuroimaging*".mp. or "functional magnetic resonance imaging".mp. or "fMRI".mp. or "BOLD imaging".mp. or "Positron emission tomography".mp. or "*FDG PET".mp. or "diffusion tensor imaging".mp. or "Magnetoencephalography".mp. or "EEG".mp. or "electroencephalograph*".mp. or "MRI".mp. or "DTI".mp. or "MEG".mp. or "PET".mp. | 1386681 |
| 4 | exp chordata, nonvertebrate/ or exp amphibians/ or exp birds/ or exp fishes/ or exp afrotheria/ or exp artiodactyla/ or exp carnivora/ or exp cetacea/ or exp chiroptera/ or exp eulipotyphla/ or exp lagomorpha/ or exp pangolins/ or exp perissodactyla/ or exp cercopithecidae/ or exp gorilla gorilla/ or exp neanderthals/ or exp pan paniscus/ or exp pan troglodytes/ or exp pongo/ or exp hylobatidae/ or exp platyrrhini/ or exp tarsii/ or exp strepsirhini/ or exp rodentia/ or exp scandentia/ or exp xenarthra/ or exp marsupialia/ or exp monotremata/ or exp reptiles/ or exp invertebrates/ or "monkey".mp. or "primate".mp. or "canine".mp. or "mouse".mp. or "rat".mp. or "rodent".mp. | 6547970 |
| 5 | ("36672026" or "36706511" or "34716192" or "34243154").ui. | 4 |
| 6 | 1 and 2 and 3 | 142 |

**Supplementary Table S2**: Embase database query, submitted July 5, 2024.

| 1 | obsessive compulsive disorder/ or "OCD".mp. or "obsessive-compulsive disorder".mp. or "obsessive compulsive disorder".mp. | 41681 |
| --- | --- | --- |
| 2 | brain surgery/ or exp psychosurgery/ or exp stereotaxic surgery/ or exp subcaudate tractotomy/ or "psychosurgery".mp. or "gamma knife".mp. or "radiofrequency*".mp. or "cingulotomy".mp. or "capsulotomy".mp. or "*leucotomy".mp. or "*tractotomy".mp. or "MRgFUS".mp. or "focused ultrasound".mp. or "focused-ultrasound".mp. or "gamma knife*".mp. or "GKR".mp. or "cyber knife".mp. or "Laser interstitial thermal therapy".mp. or "LITT".mp. or "*ablative".mp. or "*ablation".mp. | 330755 |
| 3 | neuroimaging/ or "*neuroimaging*".mp. or "functional magnetic resonance imaging".mp. or "fMRI".mp. or "BOLD imaging".mp. or "Positron emission tomography".mp. or "*FDG PET".mp. or "diffusion tensor imaging".mp. or "Magnetoencephalography".mp. or "EEG".mp. or "electroencephalograph*".mp. or "MRI".mp. or "DTI".mp. or "MEG".mp. | 1282435 |
|  | 1 and 2 and 3 | 286 |

**Supplementary Table S3**: Scopus database query, submitted July 5, 2024.

| 1 | ( TITLE-ABS-KEY ( "obsessive-compulsive disorder" OR "OCD" OR "obsessive compulsive disorder" ) ) AND ( TITLE-ABS-KEY ( "ablation techniques" OR "psychosurgery" OR "stereotaxic techniques" OR "radiosurgery" OR "psychosurgery" OR "gamma knife" OR "radiofrequency*" OR "cingulotomy" OR "capsulotomy" OR "leucotomy" OR "tractotomy" OR "MRgFUS" OR "focused ultrasound" OR "GKR" OR "cyber knife" OR "Laser interstitial thermal therapy" OR "LITT" OR "ablative" OR "ablation" ) ) AND ( TITLE-ABS-KEY ( "tomography, emission-computed" OR "positron-emission tomography" OR "positron emission tomography computed tomography" OR "single-photon tomography" OR "x-ray computed tomography" OR "magnetic resonance imaging" OR "diagnostic techniques, neurological" OR "magnetoencephalography" OR "neuroimaging" OR "functional magnetic resonance imaging" OR "fMRI" OR "BOLD imaging" OR "Positron emission tomography" OR "FDG PET" OR "diffusion tensor imaging" OR "Magnetoencephalography" OR "EEG" OR "electroencephalograph*" OR “MRI” OR “DTI” OR “MEG”) ) | 308 |
| --- | --- | --- |

**Supplementary Table S4**: The number of patients (*n*) summed across capsulotomy studies that included each ROI in a significant postoperative finding (accompanies Figure 4a). Abbreviations: HO-Cort, Harvard-Oxford cortical atlas; HO-Sub, Harvard-Oxford subcortical atlas; JHU, Johns Hopkins University DTI white matter atlas.

| **Atlas** | **Region name** | **Laterality** | ***n*** |
| --- | --- | --- | --- |
| HO-Cort | Cingulate gyrus, anterior division | Left | 248 |
| HO-Cort | Frontal orbital cortex | Right | 229 |
| HO-Sub | Caudate | Right | 228 |
| HO-Cort | Frontal orbital cortex | Left | 224 |
| HO-Sub | Caudate | Left | 214 |
| HO-Cort | Cingulate gyrus, anterior division | Right | 208 |
| HO-Sub | Thalamus | Left | 106 |
| HO-Sub | Thalamus | Right | 98 |
| HO-Cort | Inferior frontal gyrus, pars triangularis | Left | 66 |
| HO-Sub | Accumbens | Left | 63 |
| HO-Sub | Accumbens | Right | 63 |
| HO-Cort | Postcentral gyrus | Left | 52 |
| HO-Cort | Precentral gyrus | Left | 52 |
| HO-Cort | Frontal pole | Right | 44 |
| HO-Cort | Superior frontal gyrus | Right | 39 |
| HO-Cort | Inferior frontal gyrus, pars triangularis | Right | 39 |
| HO-Cort | Superior frontal gyrus | Left | 39 |
| HO-Cort | Insular cortex | Right | 39 |
| HO-Cort | Supramarginal gyrus, anterior division | Right | 31 |
| HO-Cort | Middle temporal gyrus, posterior division | Right | 31 |
| JHU | Anterior corona radiata | Right | 31 |
| JHU | Anterior corona radiata | Left | 31 |
| HO-Cort | Paracingulate gyrus | Right | 31 |
| HO-Cort | Paracingulate gyrus | Left | 31 |
| Cerebellum | Left crus I | Left | 31 |
| HO-Cort | Middle temporal gyrus, posterior division | Left | 31 |
| Cerebellum | Right crus I | Right | 31 |
| HO-Cort | Middle frontal gyrus | Left | 31 |
| HO-Cort | Cingulate gyrus, posterior division | Right | 31 |
| HO-Cort | Frontal pole | Left | 31 |
| HO-Cort | Insular cortex | Left | 31 |
| HO-Cort | Middle frontal gyrus | Right | 31 |
| Cerebellum | Right crus II | Right | 21 |
| HO-Cort | Lingual gyrus | Right | 21 |
| HO-Cort | Precuneous cortex | Left | 13 |
| Cerebellum | Vermis VI | Left | 13 |
| HO-Cort | Angular gyrus | Right | 13 |
| Cerebellum | Left crus II | Left | 13 |
| HO-Cort | Postcentral gyrus | Right | 13 |
| HO-Cort | Occipital fusiform gyrus | Right | 13 |
| HO-Cort | Middle temporal gyrus, temporooccipital part | Right | 13 |
| HO-Cort | Precuneous cortex | Right | 13 |
| HO-Cort | Precentral gyrus | Right | 8 |
| HO-Cort | Lateral occipital cortex, superior division | Right | 8 |
| HO-Cort | Inferior frontal gyrus, pars opercularis | Right | 8 |
| HO-Cort | Inferior frontal gyrus, pars opercularis | Left | 8 |
| HO-Cort | Heschl's gyrus | Right | 8 |
| HO-Cort | Heschl's gyrus | Left | 8 |
| HO-Cort | Lingual gyrus | Left | 8 |
| HO-Cort | Planum temporale | Right | 8 |
| HO-Cort | Planum temporale | Left | 8 |
| HO-Cort | Occipital pole | Right | 8 |
| Cerebellum | Right VI | Right | 8 |

**Supplementary Table S5**: The number of patients (*n*) summed across cingulotomy studies that included each ROI in a significant postoperative finding (accompanies Figure 4b). Abbreviations: HO-Cort, Harvard-Oxford cortical atlas; HO-Sub, Harvard-Oxford subcortical atlas.

| **Atlas** | **Region name** | **Laterality** | ***n*** |
| --- | --- | --- | --- |
| HO-Cort | Cingulate gyrus, anterior division | Left | 52 |
| HO-Cort | Cingulate gyrus, anterior division | Right | 52 |
| HO-Cort | Cingulate gyrus, posterior division | Right | 9 |
| HO-Cort | Temporal fusiform cortex, posterior division | Left | 9 |
| HO-Sub | Caudate | Left | 9 |
| HO-Sub | Caudate | Right | 9 |
| HO-Cort | Frontal orbital cortex | Right | 8 |

**Supplementary Table S6**: Normalized directionality index values for regions exhibiting volumetric change post-capsulotomy (accompanies Figure 5a). Abbreviations: HO-Cort, Harvard-Oxford cortical atlas; HO-Sub, Harvard-Oxford subcortical atlas; JHU, Johns Hopkins University DTI white matter atlas.

| **Atlas** | **Region name** | **Laterality** | **Normalized directionality index** |
| --- | --- | --- | --- |
| HO-Cort | Frontal orbital cortex | Right | -0.07975 |
| HO-Sub | Accumbens | Right | -0.08282 |
| HO-Sub | Accumbens | Left | -0.08282 |
| Cerebellum-MNIflirt | Left crus I | Left | -0.09509 |
| HO-Cort | Middle frontal gyrus | Right | -0.09509 |
| HO-Cort | Supramarginal gyrus, anterior division | Right | -0.09509 |
| HO-Cort | Superior frontal gyrus | Right | -0.09509 |
| HO-Cort | Superior frontal gyrus | Left | -0.09509 |
| HO-Cort | Precentral gyrus | Left | -0.09509 |
| HO-Cort | Postcentral gyrus | Left | -0.09509 |
| HO-Cort | Paracingulate gyrus | Right | -0.09509 |
| HO-Cort | Paracingulate gyrus | Left | -0.09509 |
| Cerebellum-MNIflirt | Right crus I | Right | -0.09509 |
| HO-Cort | Middle temporal gyrus, posterior division | Left | -0.09509 |
| HO-Cort | Middle temporal gyrus, posterior division | Right | -0.09509 |
| HO-Cort | Middle frontal gyrus | Left | -0.09509 |
| HO-Cort | Frontal pole | Left | -0.09509 |
| HO-Cort | Cingulate gyrus, anterior division | Left | -0.09509 |
| HO-Cort | Cingulate gyrus, anterior division | Right | -0.09509 |
| HO-Cort | Insular cortex | Right | -0.09509 |
| HO-Cort | Frontal orbital cortex | Left | -0.09509 |
| HO-Cort | Cingulate gyrus, posterior division | Right | -0.09509 |
| HO-Cort | Frontal pole | Right | -0.09509 |
| HO-Cort | Inferior frontal gyrus, pars triangularis | Left | -0.09509 |
| HO-Cort | Inferior frontal gyrus, pars triangularis | Right | -0.09509 |
| HO-Cort | Insular cortex | Left | -0.09509 |
| HO-Sub | Caudate | Left | -0.19325 |
| HO-Sub | Caudate | Right | -0.19325 |
| HO-Sub | Thalamus | Right | -0.19325 |
| HO-Sub | Thalamus | Left | -0.21779 |
| JHU-Labels | Anterior limb of internal capsule | Right | -1 |
| JHU-Labels | Anterior limb of internal capsule | Left | -1 |

**Supplementary Table S7:** Normalized directionality index values for regions exhibiting change in glucose metabolism post-capsulotomy (accompanies Figure 5b). Abbreviations: HO-Cort, Harvard-Oxford cortical atlas; HO-Sub, Harvard-Oxford subcortical atlas; JHU, Johns Hopkins University DTI white matter atlas.

| **Atlas** | **Region name** | **Laterality** | **Normalized directionality index** |
| --- | --- | --- | --- |
| HO-Cort | Precentral gyrus | Left | 0.139073 |
| HO-Cort | Lingual gyrus | Right | 0.139073 |
| HO-Cort | Postcentral gyrus | Left | 0.139073 |
| HO-Cort | Occipital fusiform gyrus | Right | 0.086093 |
| Cerebellum-MNIflirt | Vermis VI | Left | 0.086093 |
| HO-Cort | Angular gyrus | Right | 0.086093 |
| HO-Cort | Precuneous cortex | Right | 0.086093 |
| HO-Cort | Precuneous cortex | Left | 0.086093 |
| HO-Cort | Postcentral gyrus | Right | 0.086093 |
| HO-Cort | Middle temporal gyrus, temporooccipital part | Right | 0.086093 |
| HO-Cort | Lingual gyrus | Left | 0.05298 |
| HO-Cort | Occipital pole | Right | 0.05298 |
| Cerebellum-MNIflirt | Right VI | Right | 0.05298 |
| HO-Cort | Heschl's gyrus | Left | 0.05298 |
| HO-Cort | Lateral occipital cortex, superior division | Right | 0.05298 |
| HO-Cort | Heschl's gyrus | Right | 0.05298 |
| HO-Cort | Planum temporale | Right | 0.05298 |
| HO-Cort | Precentral gyrus | Right | 0.05298 |
| HO-Cort | Planum temporale | Left | 0.05298 |
| JHU-Labels | Anterior limb of internal capsule | Right | -0.03311 |
| JHU-Labels | Anterior limb of internal capsule | Left | -0.03311 |
| HO-Cort | Insular cortex | Right | -0.05298 |
| HO-Cort | Superior frontal gyrus | Left | -0.05298 |
| HO-Cort | Superior frontal gyrus | Right | -0.05298 |
| HO-Sub | Thalamus | Left | -0.05298 |
| HO-Sub | Thalamus | Right | -0.05298 |
| HO-Cort | Frontal pole | Right | -0.08609 |
| Cerebellum-MNIflirt | Left crus II | Left | -0.08609 |
| Cerebellum-MNIflirt | Right crus II | Right | -0.13907 |
| HO-Cort | Frontal orbital cortex | Right | -0.86093 |
| HO-Cort | Frontal orbital cortex | Left | -0.86093 |
| HO-Cort | Cingulate gyrus, anterior division | Right | -0.88079 |
| HO-Sub | Caudate | Right | -0.91391 |
| HO-Cort | Cingulate gyrus, anterior division | Left | -0.96689 |
| HO-Sub | Caudate | Left | -1 |

**References**

1. Waskom M. seaborn: statistical data visualization. JOSS. 2021 Apr 6;6(60):3021.
